# Novel polymyxin resistance gene family *mcr-12* from environmental *Pigmentiphaga litoralis*

**DOI:** 10.1038/s41467-026-75587-4

**Published:** 2026-07-15

**Authors:** Brodie F. Gillieatt, Ram P. Maharjan, Joel A. Cain, Nicolle H. Packer, Ruth N. Zadoks, Nicholas V. Coleman, Michael A. Kertesz, Amy K. Cain

**Affiliations:** 1 School of Natural Sciences, Faculty of Science and Engineering, Macquarie University, Sydney, NSW Australia; 2 School of Life and Environmental Sciences, Faculty of Science, The University of Sydney, Sydney, NSW Australia; 3 ARC Centre of Excellence in Synthetic Biology, School of Natural Sciences, Macquarie University, Sydney, NSW Australia; 4 Sydney School of Veterinary Science, Faculty of Science, The University of Sydney, Sydney, NSW Australia; 5 Sydney Infectious Diseases Institute, Faculty of Medicine and Health, The University of Sydney, Sydney, NSW Australia; 6 Present Address: ARC Centre of Excellence in Synthetic Biology, School of Natural Sciences, Macquarie University, Sydney, NSW Australia

**Keywords:** Antibiotics, Bacterial genes, Antimicrobial resistance, Bacteria

## Abstract

The emergence of mobile colistin resistance (*mcr*) genes threatens the efficacy of polymyxins as last-resort antibiotics for treating multidrug-resistant bacterial infections. Here, we identify a novel environmental *mcr* gene, *mcr-12*, discovered in *Pigmentiphaga litoralis* from Australian sediment, and evaluate its potential role in the intersection between environmental resistance reservoirs and clinically relevant bacteria. Gene *mcr-12* was located within a metal-resistance gene cluster on plasmid pPLE30.2, which also carried a predicted novel β-lactamase gene (*bla*_OXA-1383_). Removal of pPLE30.2 increased polymyxin susceptibility 32-fold, while reintroduction of *mcr-12* restored resistance. Despite its low amino-acid identity to known MCR enzymes, MCR-12 confers polymyxin resistance by phosphoethanolamine transferase modification of lipid A. Expression of *mcr-12* in *Pseudomonas* spp. and *Acinetobacter baumannii* conferred polymyxin resistance, suggesting that *mcr-12* is compatible between environmental strains and clinical pathogens. The discovery of a new *mcr* gene from an environmental source and from outside Gammaproteobacteria highlights the need for further surveillance efforts within a One Health framework.

## Abstract (web summary)

Multiple families of transferable *mcr* genes drive bacterial resistance to polymyxin antibiotics. Here, Gillieatt et al. identify a new type of plasmid-borne *mcr* gene in an environmental bacterium and show that it confers polymyxin resistance in the native microbe and when transferred to other bacteria.

## Introduction

Antimicrobial resistance (AMR) is a major health issue, with 4.71 million deaths linked to AMR in 2021 globally^1^. Most AMR deaths are caused by the seven hospital-associated ESKAPEE bacteria (*Enterococcus faecium*, *Staphylococcus aureus*, *Klebsiella pneumoniae*, *Acinetobacter baumannii*, *Pseudomonas aeruginosa*, *Enterobacter* spp., and *Escherichia coli*)^1,2^. With a diminishing supply of effective antibiotics and limited prospects of developing new antibiotics, the once undesirable antibiotic class of polymyxins are experiencing a resurgence in use^3^. Polymyxins B and E (colistin) are reserved for last-resort treatment of multidrug-resistant infections, owing to their relatively low level of resistance, but also their potential for neurotoxicity and nephrotoxicity^3^. Polymyxin antibiotics are bactericidal against Gram-negative bacteria by displacing divalent cations ionically bound to the lipid A phosphate component of the lipopolysaccharide or lipooligosaccharide, thereby destabilising outer membrane integrity^4^.

Polymyxin resistance mechanisms have been primarily determined for Enterobacteriaceae, with less focus on other critical pathogens like *P. aeruginosa* and *A. baumannii*^5^. The most common polymyxin resistance mechanism in Enterobacteriaceae involves the targeted modification of the lipid A with cationic residues such as phosphoethanolamine (PEtN) and 4-amino-4-deoxy-L-arabinose (L-Ara4N)^6,7^. These modifications disrupt the electrostatic interaction between polymyxin and lipid A, preventing antibiotic binding. Polymyxin resistance was traditionally considered to be mediated by non-transferable and potentially unstable^8^ mutations in transcriptional regulators of chromosomal PEtN transferases and L-Ara4N transferase genes^3,8,9^.

In the last decade, a new class of transferable resistance genes that encode PEtN transferases, the mobile colistin resistance (*mcr*) genes, has taken on a major role in polymyxin resistance spread. The first description of *mcr-1* in *E. coli* from Chinese swine farms in 2015^10^ disproved the widely held assumption that polymyxin resistance was not transferable. Since then, *mcr* genes have been found worldwide in numerous Gammaproteobacteria, various plasmid types, and associated with sources including humans, animals, and meat products (Supplementary Table 1). To date, eleven *mcr* families have been categorised (*mcr-1* up to *mcr-10*^11^, with *mcr-11* currently pending publication (WP_150870284)). Studies have focused exclusively on *mcr* genes from clinical, meat products, or livestock sources, and all *mcr* families have been isolated from Enterobacteriaceae, although reservoirs of these genes may exist in more diverse taxa^12,13^. To appreciate the extent of mobile polymyxin resistance determinants within a One Health framework, it is important to characterise the diversity of *mcr* genes present in environmental reservoirs. This provides critical context for gene dissemination and may inform which resistance determinants should be prioritised for future surveillance.

Here, we describe and characterise *mcr-12*, a novel, plasmid-borne polymyxin resistance gene from an environmental isolate of *Pigmentiphaga litoralis*, a Betaproteobacterium recovered from heavy metal-contaminated sediment from the Central Coast, NSW, Australia. We characterised the genomic context and phylogeny of *mcr-12* and tested the hypothesis that MCR-12 confers polymyxin resistance in *P. litoralis* and a range of alternative hosts, including ESKAPEE pathogens and model organisms. This study provides rare insights into a new potential genetic reservoir for last-resort antibiotic resistance in the environment that has not yet spread to clinically relevant pathogens and may be driven by heavy metal selection.

## Results

### *mcr-12* is carried by a multiple metal resistance plasmid in *Pigmentiphaga litoralis*

A yellow pigmented isolate (E30), tolerant to at least 0.5 mM cadmium acetate in R2A agar, was cultured from heavy metal contaminated freshwater sediment as part of a broader assessment of Australian heavy metal and antibiotic resistant environmental isolates. Whole genome sequencing and alignment of the entire 16S gene revealed 99.66% identity to *P. litoralis* JSM 061001 (GenBank: NR_044530.1).

**Fig. 1:**
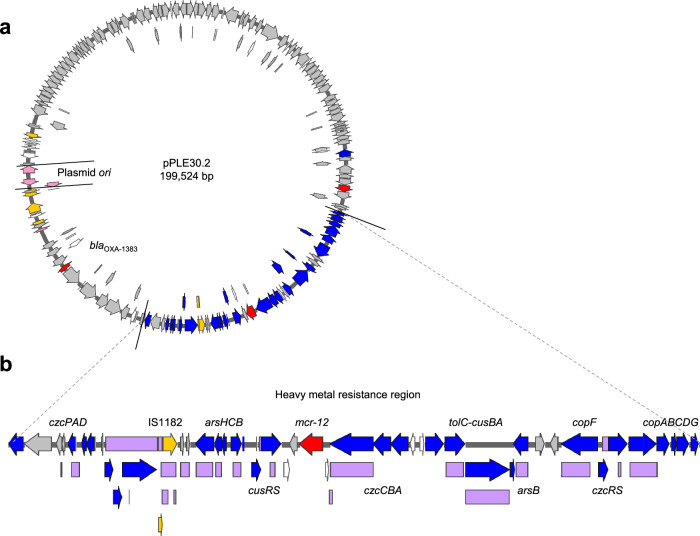
Plasmid gene map of pPLE30.2. **a** Map of plasmid pPLE30.2 and **b** an exploded view of the resistance region of pPLE30.2 that includes *mcr-12*. Arrows indicate bioinformatically predicted genes. Blue colour indicates metal resistance genes, red indicates antibiotic resistance genes, yellow represents mobile genetic elements, pink represents plasmid maintenance genes including origin of replication (*ori*), grey represents non-resistance related genes, and white represents hypothetical genes of unknown identity. Violet bars represent DNA alignments. Map created in SnapGene.

Whole genome sequencing of two independent colonies of *P. litoralis* E30 revealed the presence of a 199.5 kb plasmid, which was named pPLE30.2 (Fig. 1a; GenBank accession number: PQ035968). Plasmid pPLE30.2 harbours a 50 kb metal resistance region, featuring genes homologous to *arsHCB* (arsenic resistance)^14^, *cusAB-tolC* (copper resistance)^15^, *czcCBA* (cadmium/zinc/cobalt resistance)^16^ and *copABCDG* (copper resistance)^17^ (Fig. 1b). The configuration of this heavy metal resistance region is novel, as there was negligible identity in the inter-gene backbone to any known sequence. Although no complete insertion sequence elements or prophages could be detected in pPLE30.2, one intact IS*1182* transposase gene was identified within the metal resistance region, which could indicate movement events. The plasmid does not belong to a traditional incompatibility group and could not be mobilised into a plasmid-cured strain of *P. litoralis* by biparental conjugation or triparental conjugation using helper plasmid pRK2013.

Manual inspection of the heavy-metal resistance region revealed a novel potential resistance gene located between *czcCBA* and *cusRS* (Fig. 1b; bases 130397–132043) with its product displaying 39.3% amino acid identity compared to the polymyxin resistance determinant EptA from *Salmonella enterica* (GenBank: P36555.1) (Table 1). This novel gene was not detected by screening with an antimicrobial resistance database, but its annotation, provided by Prokka and manual BLASTP alignment, was intriguing as EptA is normally harboured on the chromosome^18,19^. Analysis of the *P. litoralis* E30 chromosome revealed an *eptA* and *eptC* gene, however the respective protein sequences had low identity to the plasmid-borne homologue (Table 1). This suggests that the novel plasmid-borne homologue is of a distinct gene lineage from that of the chromosomal *eptA*.

**Table 1:** Potential novel phosphoethanolamine (PEtN) transferase genes in the *Pigmentiphaga litoralis* genome were aligned using the Comprehensive Antibiotic Resistance Database (CARD), SwissProt, or the NCBI non-redundant protein sequences database

*P. litoralis* protein	Reference database for alignment	Reference protein (GenBank accession)	Identity (%)	Similarity (%)	Query coverage (%)	*E-*value
MCR-12	SwissProt	EptA (P36555.1)	39.30	57.93	98.91	8e^-128^
MCR-12	non-redundant	Uncharacterised PEtN transferase (WP_132257985)	57.78	74.26	98.54	0
MCR-12	CARD	MCR-5.1 (ASK40551.1)	37.62	57.73	97.99	2e^-119^
MCR-12	CARD	MCR-5.2 (AVM85875.1)	37.62	57.73	97.99	6e^-118^
EptA	SwissProt	EptA (P36555.1)	33.58	52.55	97.51	4e^-103^
EptA	non-redundant	Uncharacterised PEtN transferase (WP_183026766.1)	56.10	71.22	97.69	0
EptA	*P. litoralis* genome	MCR-12	40.57	57.17	86.68	1e^-115^
EptC	SwissProt	EptC (P0CB39.1)	26.86	44.38	100	4e^-47^
EptC	non-redundant	Uncharacterised PEtN transferase (WP_179586969.1)	96.47	97.84	100	0
EptC	*P. litoralis* genome	MCR-12	26.32	42.41	63.21	1e^-17^

Similarity refers to amino acids that are either identical or equivalent to a conservative missense mutation in the query protein.

**Fig. 2:**
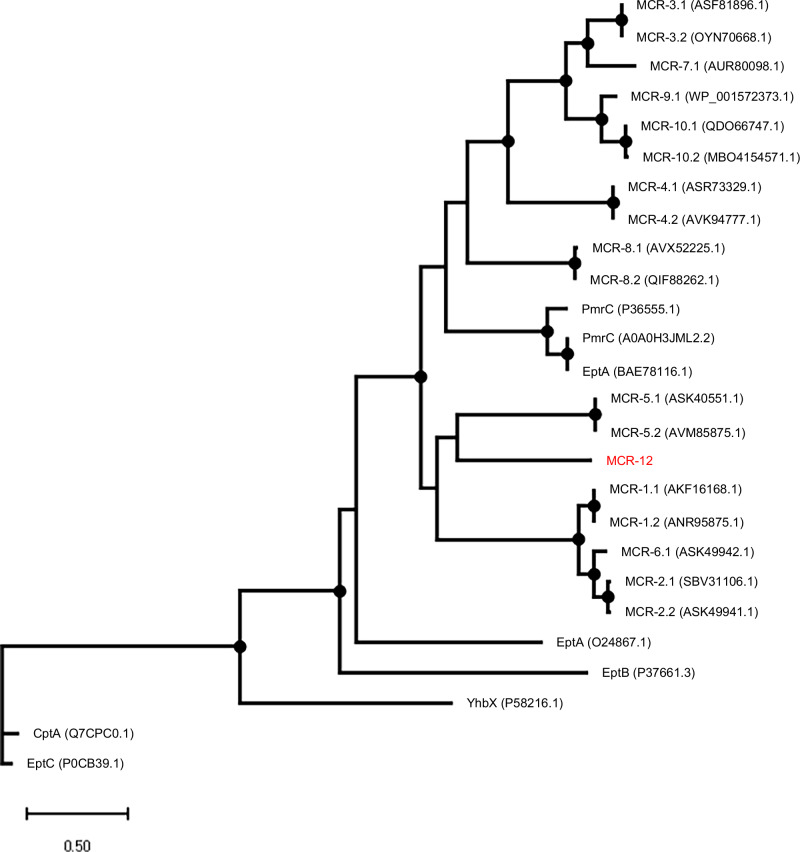
Unrooted maximum likelihood phylogeny tree displaying MCR-12 as a novel phosphoethanolamine transferase. Protein entries confined to SwissProt entry alignments with an identity to MCR-12 (in red font) with *E*-value < 1 × 10^-6^, and two representatives from each MCR family from the Comprehensive Antibiotic Resistance Database. Filled circle indicates > 85% bootstrap replications. Source data are provided as a Source Data file.

**Fig. 3:**
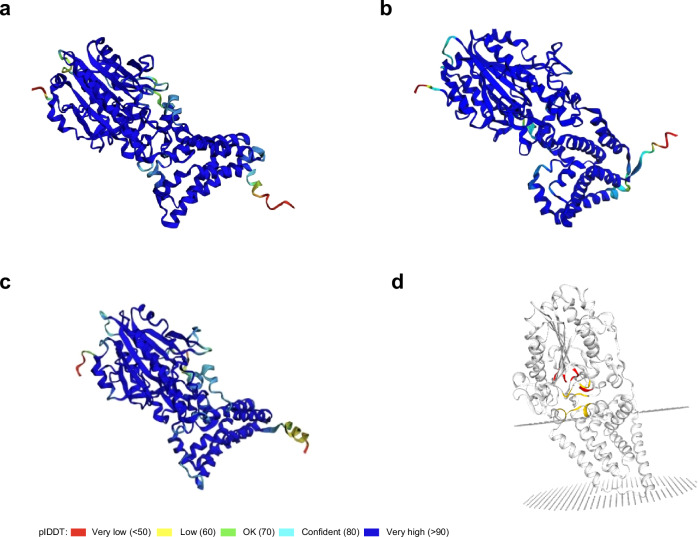
MCR-12 displays a conserved phosphoethanolamine transferase structure. Predicted model of **a** *Pigmentiphaga litoralis* MCR-12 (GenBank: XFF02116.1), **b** *Salmonella enterica* EptA (GenBank: P36555.1), and **c** *Escherichia coli* MCR-5.2 (GenBank: AVM85875.1) constructed using AlphaFold2^78^. Coloured residues indicate confidence of predicted local distance difference test (pIDDT) for panels (**a**–**c**) only. **d** SWISS-MODEL homology model of *P. litoralis* MCR-12 in cartoon view with active site residues in yellow and zinc binding residues in red.

Further phylogenetic analysis of evolutionary distances of homologous proteins in the SwissProt and CARD databases revealed that the novel protein was more closely related to MCR-5.1 from *S. enterica* (GenBank: ASK40551.1) and MCR-5.2 from *E. coli* (GenBank: AVM85875.1) than to EptA from *S. enterica* (Fig. 2, Supplementary Fig. 1). When the predicted structure of the novel protein (Fig. 3a) was superimposed onto *S. enterica* EptA (Fig. 3b) and *E.* *coli* MCR-5.2 (Fig. 3c) it aligned tightly, despite the low amino acid similarity (Table 1). The predicted structure included five transmembrane α helices, a cell-exterior domain containing β sheets, and an active cleft containing the key functional residues responsible for phosphatidylethanolamine binding and zinc binding in MCR enzymes^18^ (Fig. 3d, Supplementary Fig. 2). The novel gene was assigned the name *mcr-12* (GenBank: XFF02116.1), the first of a new *mcr* family. This naming is justified by its predicted structure and location on pPLE30.2, as *mcr* genes are carried on mobile genetic elements^18^.

### *mcr-12* confers functional polymyxin resistance in *Pigmentiphaga litoralis*

The structural similarity of the *mcr-5* and *eptA* gene products implied that *mcr-12* would also confer polymyxin resistance. *P. litoralis* E30 was highly resistant to polymyxin B with a minimum inhibitory concentration (MIC) of 80 µg/ml. After curing pPLE30.2 from *P. litoralis* E30, the MIC was reduced 32-fold to only 2.5 µg/ml, indicating that the plasmid harbouring *mcr-12* conferred polymyxin resistance (Table 2). The expression of *mcr-12* in pPLE30.2 was not significantly changed after exposure to polymyxin B, over a concentration range of 0.02–2 µg/mL. The constitutive expression of *mcr-12* was confirmed by reverse transcription-quantitative PCR (RT-qPCR) with the *mcr-12* transcripts normalised to a chromosomal housekeeping gene (*rpoB*; Supplementary Fig. 3a), or a plasmid copy number control (*repB*; Supplementary Fig. 3b). Further, plasmid curing resulted in 4–32-fold reductions in tolerance to zinc, arsenite, and arsenate (Table 2), indicating that genes on pPLE30.2 also mediated heavy metal tolerance. Plasmid curing resulted in a smaller two-fold decrease in tolerance to both cobalt and cadmium (MIC reduced from 2 mM for the plasmid-carrying strain to 1 mM for the plasmid-cured strain), and copper tolerance remained unchanged (MIC of 5 mM).

**Table 2:** Minimum inhibitory concentration (MIC) of polymyxin B (µg/mL) and heavy metals (mM) for *Pigmentiphaga litoralis* plasmid-carrying and plasmid-cured strains lacking pPLE30.2

Antimicrobial	Plasmid-carrying MIC	Plasmid-cured MIC	Plasmid-carrying vs plasmid-cured MIC fold-reduction	Relevant plasmid gene(s)
**Polymyxin B**	80	2.5	32	*mcr-12*
**Arsenate**	128	4.0	32	*arsHCB*
**Arsenite**	5.0	0.2	32	*arsHCB*
**Zinc**	8.0	2.0	4	*czcCBA*

MICs represent consensus from multiple independent experiments (*n* = 3 (polymyxin B) or *n* = 2 (metals), each with 2–4 technical replicates) where MIC is OD_600_ < 0.1 after 48 h growth in cation-adjusted Mueller-Hinton broth. Source data are provided as a Source Data file.

**Fig. 4:**
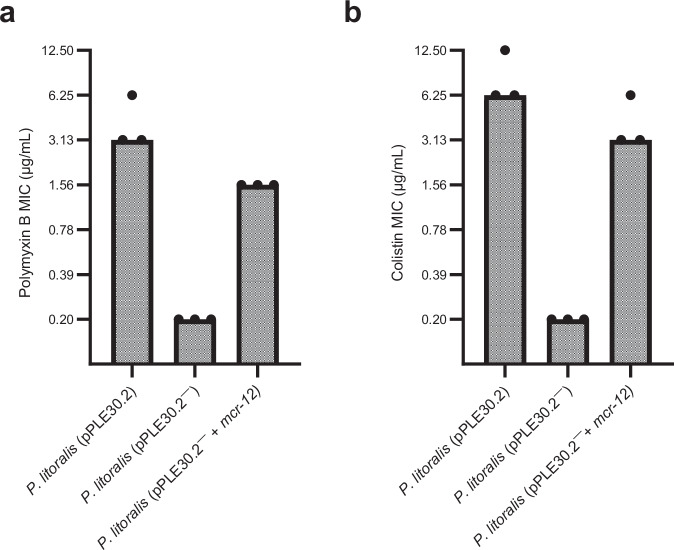
The *mcr-12* gene contributes resistance to polymyxin B and colistin in *Pigmentiphaga litoralis.* Minimum inhibitory concentrations (MICs) of **a** polymyxin B and **b** colistin were determined after 96 h using the Biolog Odin L system. MICs were measured for the wild-type strain carrying the *mcr-12*-harbouring plasmid pPLE30.2, a plasmid-cured isogenic strain lacking pPLE30.2 (pPLE30.2^—^), and the plasmid-cured strain complemented with *mcr-12* on pBBR1MCS-2-*mcr-12* (pPLE30.2^—^ + *mcr-12*). Individual points represent independent biological replicates (*n* = 3, each with 2–4 technical replicates). Columns indicate median MIC values. Source data are provided as a Source Data file.

To further investigate the role of *mcr-12* in mediating polymyxin resistance, we cloned *mcr-12* into the pBBR1MCS-2 broad host range expression plasmid^20^. After the re-introduction of *mcr-12* into the plasmid-cured *P. litoralis* strain, the polymyxin resistance phenotype was restored, yielding an 8- and 16-fold increase in the MICs for polymyxin B and colistin, respectively (Fig. 4). Collectively, these results confirm that *mcr-12* confers polymyxin resistance in *P. litoralis*.

Outside the metal resistance region on pPLE30.2, another potential resistance gene was identified using the CARD database. It encoded a protein with 59% amino acid identity to OXA-926, an oxacillinase from *K. pneumoniae* (GenBank: WP_114268491.1)^21^. This novel class D β-lactamase was assigned the name OXA-1383 (GenBank: WP_407792312). *P. litoralis* E30 displayed low phenotypic susceptibility to high concentrations of ampicillin, carbenicillin, piperacillin, cephalothin, and cefepime (Supplementary Table 2). However, susceptibility of *P. litoralis* E30 to ten β-lactam antibiotics was not altered by loss of pPLE30.2, nor was resistance gained when *bla*_OXA-1383_ was expressed in *E. coli* TOP10 (a DH10b derivative hereafter simplified to *E. coli*), as tested by MIC or the modified Stokes’ method of disc diffusion (Supplementary Table 3).

### *mcr-12* confers polymyxin resistance in ESKAPEE pathogens and related bacteria

The breadth of *mcr-12* activity was assessed in a range of diverse and clinically relevant species, using the same or similar expression systems to the one described above. Expression plasmids containing *mcr-12* were transferred to representatives of clinically important ESKAPEE pathogens (*K. pneumoniae, A. baumannii*, *P. aeruginosa, Enterobacter cloacae*, and *E. coli*) and polymyxin B susceptibility was evaluated by MIC. *P. aeruginosa* PAO1 and *A. baumannii* ATCC 17978 containing *mcr-12* displayed a two-fold increase in MIC, compared to their respective vector controls (Table 3, Supplementary Fig. 4a, d). The level of resistance conferred by *mcr-12* was identical to that of *mcr-1*, which was utilised as a reference for clinically relevant *mcr* genes. No obvious change in MIC was observed for *E. coli* expressing *mcr-12*, although expression of *mcr-1* produced the anticipated increase in MIC to 4 µg/mL^10^ (Table 3). Similarly, *mcr-12* did not alter the MIC for *K. pneumoniae*, *E. cloacae*, and uropathogenic *E. coli*. This indicates that *mcr-12* could be less effective in conferring polymyxin resistance in Enterobacteriaceae.

**Table 3:** Polymyxin B minimum inhibitory concentration (MIC)^†^ (µg/mL) increased for some strains expressing *mcr-12*. Gene *mcr-12* or *mcr-1* cloned into expression vectors pBBR1MCS-2, pBBR1MCS-5, or pVLT33. Consensus of *n* = 3, apart from *n* = 6 for *Pseudomonas aeruginosa* vector control and vector with *mcr-12*. Each *n* consists of 2–4 technical replicates. NT = not tested

Strain (vector)	Vector control	Vector with *mcr-12*	MIC fold change^‡^	Vector with *mcr-1*
***Acinetobacter baumannii*** **ATCC 17978 (pVLT33)**	1.0	2.0	2	2.0
***Acinetobacter baumannii*** **BAL062 (pVLT33)**	1.0	2.0	2	8.0
***Acinetobacter baumannii*** **Ex003 (pVLT33)**	1.0	2.0	2	8.0
***Pseudomonas aeruginosa*** **PAO1 (pBBR1MCS-5)**	1.0	2.0	2	2.0
***Pseudomonas aeruginosa*** **PA14 (pBBR1MCS-2)**	1.0	2.0	2	2.0
***Pseudomonas protegens*** **Pf-5 (pBBR1MCS-2)**	32.0	256.0	8	256.0
***Klebsiella pneumoniae*** **Ecl8 (pBBR1MCS-2)**	1.0	1.0	No change	NT
***Enterobacter cloacae*** **NCTC 9394 (pBBR1MCS-5)**	0.5	0.5	No change	NT
**Uropathogenic** ***Escherichia coli*** **NCTC 11334 (pBBR1MCS-5)**	0.5	0.5	No change	NT
***Escherichia coli*** **TOP10**	0.5	0.5	No change	4.0

Source data are provided as a Source Data file.

^†^ MIC determined by OD_590_ < 0.1 after 48 hours growth in cation-adjusted Mueller-Hinton broth, Biolog redox dye, and (*A. baumannii* strains only) 0.25 mM isopropyl ß-D-1-thiogalactopyranoside.

^‡^ Fold change of vector control vs *mcr-12*-containing insert for each strain.

Due to the clear polymyxin resistance phenotype that *mcr-12* displayed in *Acinetobacter* and *Pseudomonas*, heterologous expression was expanded to more clinical and environmental representatives of these genera. For *Pseudomonas*, *Pseudomonas protegens* Pf-5 was used as an environmental model and exhibited an MIC of 256 µg/mL (eight-fold increase over vector control) with the addition of heterologously expressed *mcr-12* (Table 3, Supplementary Fig. 4f). *P. aeruginosa* PA14 was tested as an additional clinical model and displayed the same doubling of MIC as strain PAO1 (Table 3, Supplementary Fig. 4e). *A. baumannii* was further represented by strain BAL062 as a clinical isolate of global clone 2, and strain Ex003 as an environmental isolate of global clone 1^22^. Both strains displayed similar results to strain 17978, with a doubling in MIC when transformed with *mcr-12* (Table 3, Supplementary Fig. 4b, c). In these additional *A. baumannii* strains, the MIC conferred by *mcr-1* was four-fold greater than that mediated by *mcr-12*.

### MCR-12 is a phosphoethanolamine transferase

To investigate the mechanism by which *mcr-12* confers polymyxin resistance across heterologous hosts, the gene was expressed in *E. coli*, a model organism with a well-characterised polymyxin resistance background and outer membrane structure^23^. Similar to the previous Enterobacteriaceae hosts, the polymyxin B MIC was not altered in the presence of *mcr-12* (Supplementary Fig. 5a), however, there was a two-fold increase in MIC for colistin (Supplementary Fig. 5b). RT-qPCR confirmed that *mcr-12* was transcribed at a high level in *E. coli* carrying *mcr-12* during both exponential and stationary growth phases (Supplementary Fig. 5c). The transcription of endogenous PEtN transferases genes *pmrC, eptB, eptC*, and L-Ara4N transferase gene *arnT* was unchanged by the expression of *mcr-12* (Supplementary Fig. 5d–g). These findings confirmed that MCR-12 was solely responsible for the change in colistin susceptibility.

**Fig. 5:**
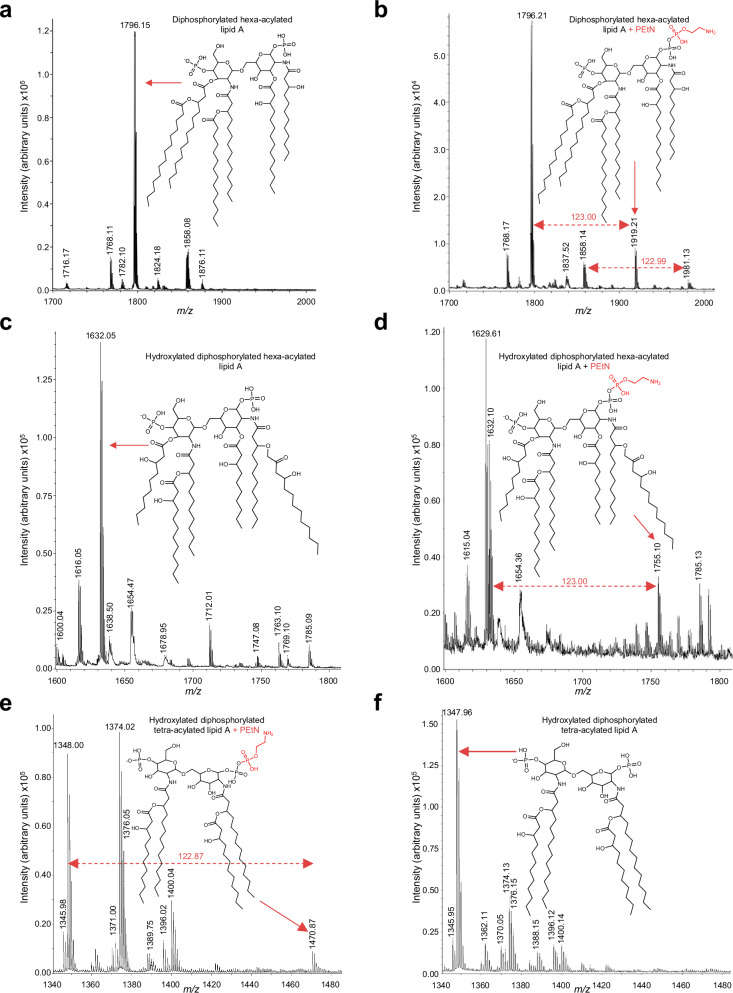
The addition of a phosphoethanolamine group to lipid A by MCR-12. Lipid A modification is revealed by matrix-assisted laser desorption/ionization-mass spectrometry profiles of lipid A extract from *Escherichia coli* TOP10 transformed with **a** pBBR1MCS-2 and **b** pBBR1MCS-2-*mcr-12*, *Pseudomonas protegens* Pf-5 transformed with **c** pBBR1MCS-2 and **d** pBBR1MCS-2-*mcr-12*, and *Pigmentiphaga litoralis* **e** plasmid-carrying and **f** plasmid-cured strains. Interpreted structures correspond to the peaks indicated by red solid arrows and are representational only, based on the mass changes to the lipid A. Dashed red lines indicate the *m/z* difference in peaks corresponding to unmodified lipid A structures and lipid A modified by phosphoethanolamine addition. Representative structures are displayed from *n* = 2 for panels (**a**–**d**) and *n* = 3 for panels (**e**, **f**). Annotated peaks have an area >1500 (**a**, **b**), >3000 (**c**, **e**, **f**), or >1000 (**d**).

All MCR enzymes confer polymyxin resistance by transferring cationic PEtN moieties onto lipid A^3^. To confirm the predicted function of MCR-12 as an active PEtN transferase, we compared the lipid A composition of a model organism that did not display an obvious polymyxin B MIC shift (*E. coli*) and a diverse host that displayed the greatest MIC increase (*P. protegens*) (Table 3). Matrix-assisted laser desorption/ionization-mass spectrometry of both species transformed with pBBR1MCS-2-*mcr-12* revealed peaks consistent with PEtN modification which were absent in the empty vector controls (pBBR1MCS-2). For *E. coli*, two 123 Da shifts corresponding to PEtN addition were observed: one on a predicted diphosphorylated hexa-acylated lipid A (*m/z* 1796 increased to *m/z* 1919)^24^, and the second on a peak of unknown structure (Fig. 5a, b; *m/z* 1858 increased to *m/z* 1981). For *P. protegens*, the PEtN group was likely added to a hydroxylated diphosphorylated hexa-acylated lipid A (Fig. 5c, d; *m/z* 1632 increased to *m/z* 1755), a lipid A structure common in *Pseudomonas*^25^. This modification aligns with the established mechanism of polymyxin resistance mediated by other *mcr* genes^11^ and consolidates the classification of *mcr-12* as a PEtN transferase.

With clear spectral evidence of PEtN modification detected in heterologous hosts, lipid A analysis was extended to the original *P. litoralis* isolate to confirm whether native expression produced the same modification. Comparison of the lipid A spectral profiles between the pPLE30.2-carrying strain and its plasmid-cured counterpart revealed a peak at *m/z* 1471 that was unique to the pPLE30.2-carrying strain. This peak is 123 Da greater than the shared *m/z* 1348 peak (Fig. 5e, f). Two shared peaks between the strains constitute the major lipid A structures. The *m/z* 1348 peak correlates with a hydroxylated diphosphorylated tetra-acylated lipid A, which is prevalent in Betaproteobacteria^26^, containing two C14 and two C12 acyl chains, while the *m/z* 1374 peak likely represents an unsaturated analogue comprising three C14 and one C12 acyl chains^26^. Thus, we confirm that the enzymatic role of MCR-12 in its native host is to modify lipid A with a PEtN moiety, while the core lipid A structure remains unchanged.

## Discussion

This study characterised a new polymyxin resistance gene, *mcr-12*, identified on a plasmid present in an environmental isolate of *P. litoralis*, a Betaproteobacterium. We show that the *mcr-12* gene can confer polymyxin B and colistin resistance to the *P. litoralis* host, but also to the clinically relevant ESKAPEE pathogens *A. baumannii* and *P. aeruginosa* and the plant pathogen *P. protegens*. We demonstrate that its mechanism of conferring polymyxin resistance is in line with that encoded by other *mcr* genes – by the addition of PEtN to the lipid A. The *mcr-12* family is the first *mcr* family discovered outside of a clinical, food, or livestock context, the first in a non-Gammaproteobacterial host, and the first in the Southern Hemisphere. The genomic context of *mcr-12* uniquely associates polymyxin resistance, multiple heavy metal tolerance, and predicted β-lactam resistance genes, indicating the possibility of co-resistance and/or co-selection. Given the critical importance of polymyxins as antibiotics of last resort, the detection of a new *mcr* gene from freshwater sediment should spur further studies examining the mobility and distribution of polymyxin resistance genes that may possess greater clinical relevance.

Historically, *mcr* genes have almost exclusively been monitored in Enterobacteriaceae^11^, with over 99% of *mcr* genes detected in *E. coli*, *S. enterica*, and *K. pneumoniae* as of 2019^5^. As a result, most of our understanding of *mcr* genes comes from studies on Enterobacteriaceae, and the role of PEtN transferases is less clear in other taxa not routinely screened for polymyxin resistance, such as Pseudomonadales^24^. Taking a broader lens to detect resistance genes in diverse species is important to properly assess the emergence of new resistance genes. We have shown that MCR-12 reduces polymyxin susceptibility to a greater degree in Pseudomonadales than in Enterobacteriaceae. Although the MIC difference observed here is modest, the consistent two-fold decrease in susceptibility across all *Acinetobacter* and *Pseudomonas* strains tested here demonstrates phenotypic reproducibility. Considering that the clinically relevant *mcr-1* produced a similar MIC shift in *Pseudomonas*, *mcr-12* may be comparably effective in conferring lipid A modification and polymyxin resistance in select strains (Table 3). The largest MIC difference conferred by the heterologous expression of *mcr-12* was 8-fold in *P. protegens*. This contrasts strongly to the unchanged polymyxin B MIC of all examined Enterobacteriaceae after addition of *mcr-12* (Table 3).

Lipid A modification with PEtN was still detected in *E. coli* despite the absence of a change in polymyxin B susceptibility phenotype. One potential factor influencing the effect of *mcr-12* on polymyxin resistance across species may be differences in cell envelope structure and modification. As expected, core lipid A structure differed among the three analysed species^25^, and these structural differences may influence the net ionic effect of PEtN addition. Alternatively, L-Ara4N modification is an effective strategy for polymyxin resistance in Enterobacteriaceae^6^, but the *arn* operon is absent in *A. baumannii*^27^. Rather, *A. baumannii* can completely remove its lipid A^28^, a strategy that has not been observed in the other species used in this study^29^. Another factor that may influence MIC changes upon heterologous expression is codon usage. Codon usage was not harmonised across host organisms because our aim was to assess the transferability of the *P. litoralis mcr-12* native coding sequence and test its resistance phenotype in clinically-relevant bacteria, thereby modelling what may be the impacts of horizontal gene transfer of *mcr-12*. Nonetheless, lipid A analysis confirmed that MCR-12 retained enzymatic functionality even in *E. coli*. It remains plausible that enzyme kinetics in the heterologous host was diminished, resulting in lower overall levels of PEtN-modified lipid A. Relative quantification of PEtN-modified lipid A may indicate whether this is the case. Finally, the high-level expression of *mcr-12* may impose a fitness cost and reduce cell viability, as in the case of *mcr-1*.*1*^30,31^. However, the impact of *mcr-12* expression on cell growth was relatively minor during the exponential growth phase and did not impact growth during the stationary phase (Supplementary Fig. 6).

The progenitor host(s) or original reservoir(s) of *mcr* genes remain unclear, as is the case for many antibiotic resistance genes on mobile elements. However, the discovery of *mcr-12* in *P. litoralis* from a sediment environment adds to a growing body of evidence supporting a diverse distribution of these resistance genes in the environment^13,32,33^. For example, *mcr* genes have been frequently found in aquatic microbes such as *Aeromonas* and *Shewanella* when sampling aquatic farms^34^, which reinforces the importance of monitoring more diverse environments for *mcr* genes. Despite its limited phenotype in some Gammaproteobacteria, MCR-12 clearly affects polymyxin sensitivity in *P. litoralis* and it would be valuable to investigate its role in other Betaproteobacteria.

Precedent has been set of transfer between environmental Betaproteobacteria and clinically relevant Enterobacteriaceae with *mcr-5.1*, the closest homologue of *mcr-12* by evolutionary distance (Fig. 2). The *mcr-5.1* gene is found in Tn*3* in the genome of *Pigmentiphaga* sp. and on the chromosome of *Cupriavidus gilardii*, either of which could have been the donor cell when Tn*3* transposed onto a plasmid which mobilised into *S. enterica*^13,32^. Concerningly, this *Pigmentiphaga* sp. carrying *mcr-5.1* was isolated from a human ear infection^35^, and colistin-resistant *Pigmentiphaga* has recently been implicated in a further ear infection^36^, which clearly enhances the clinical relevance of this genus. Another case of *mcr-1.1* being transferred between *Alcaligenes faecalis* (of the same family as *Pigmentiphaga*) and Enterobacteriaceae^33^ indicates that Betaproteobacteria deserve more attention as potential reservoirs of *mcr* genes, and ought to be monitored for mobilisation events.

The presence of *mcr-12* on a plasmid presents an opportunity for horizontal gene transfer. Previous studies have implicated insertion sequences^37–40^ and prophages^41^ in the transfer of PEtN transferase genes, including *mcr-5.1*^32^, and the construction of complex, multi-drug resistance plasmid regions^42^. Although no direct evidence of an active or complete transposable element was found on the host plasmid pPLE30.2, it did contain an incomplete IS*1182* insertion sequence downstream, which may have been involved in the original integration of metal resistance genes. Most plasmid types known to carry *mcr* are conjugative^43–45^, which facilitates the contact-dependent spread of *mcr* genes amongst bacterial hosts. The plasmid carrying *mcr-12*, pPLE30.2, contrasts with these in that it belongs to an unknown plasmid type with a backbone bearing no homology with any known genome (Fig. 1) and is likely to be non-mobilisable. Due to the novelty of the genetic backbone, it is possible that the plasmid may be mobilised by alternative helper plasmids, such as PromA plasmids^46^.

A unique feature of *mcr-12* compared to other *mcr* genes is its genomic location being directly flanked by multiple heavy metal resistance operons, including *czcCBA* and *arsHCB*, which are extremely likely to contribute to the heavy metal tolerance phenotype (Table 2). Despite their proximity, it is not suggested that they share a regulatory pathway as *mcr-12* appears constitutively expressed, consistent with *mcr-1*, but not *mcr-9*^47^, whereas it is established that *czcCBA* and *arsHCB* are regulated by CzcRS^16^ and ArsR^48^ following ion exposure, respectively. Metal and antibiotic co-resistance has been previously observed in many transposons and plasmids^49^, but our study is the first to experimentally characterise a multiple heavy metal resistance region with an *mcr* gene. One case of a Tn*3* containing *mcr-5.1* together with a single chromium efflux pump regulator gene *chrB* has been described^32^, but this gene encodes a transcriptional repressor of the chromium efflux pathway and does not confer chromium tolerance^50^. The indirect selection for *mcr-12* by maintaining the metal resistance genes on pPLE30.2 could indicate an original need for co-resistance in a highly selective environment such as metal-contaminated sediment^51^. Examples of metal and polymyxin co-selection in *E. coli* include an increase in resistance to polymyxin B following exposure to environmental concentrations of copper^52^, and L-Ara4N transferases induced by vanadate exposure^53^. Our findings support the One-Health narrative that heavy metal- or pharmaceutical-contaminated environments may be ultimately driving the emergence of antibiotic resistance.

The co-occurrence of a predicted β-lactamase gene on pPLE30.2 is consistent with other plasmids that carry *mcr-3.1*^43^, *mcr-7.1*^44^, and *mcr-5.1*^32^ and another β-lactamase gene, which may be due to the selection for multiple resistance genes. The β-lactam substrate profile of OXA-1383 appears not to include any of the ten β-lactams tested here (Supplementary Tables 2, 3), placing OXA-1383 among a growing group of homology-predicted OXA-domain enzymes for which substrate specificity remains unknown^54^. Substantial amino acid heterogeneity within the OXA family complicates functional prediction^54^; notably, the substrate profile of OXA-1383 differs from its closest known homologue, OXA-926, in respect to piperacillin and cephalothin^21^. However, the tested β-lactams cannot be excluded as potential substrates, as in vitro expression of *bla*_OXA-1383_ in *P. litoralis* may have been impaired by insufficient induction, while heterologous expression may have limited enzyme activity through inefficient protein folding and localisation. Alternatively, *bla*_OXA-1383_ may not encode a functional resistance enzyme, and its presence may be incidental and not impact the fitness of the host. The maintained high β-lactam resistance despite the removal of pPLE30.2 may be due to alternative β-lactamases, efflux pumps^55^, or modified penicillin-binding proteins^56^.

Overall, the discovery of *mcr-12* in a *P. litoralis* environmental isolate advances our understanding of the activity and diversity of polymyxin resistance genes from the environment. Assessing resistance gene reservoirs is an important but developing field. Considering that *mcr-1* was discovered only a decade ago, and before that it was widely believed that polymyxin resistance was not transferable, more *mcr* genes await detection and characterisation. The discovery of *mcr-12* outside of a polymyxin-exposed setting also implies that *mcr* genes may be more widespread than previously thought. This vastly increases the opportunities for pathogens to acquire polymyxin resistance genes from anthropogenically contaminated reservoirs, and demonstrates the importance of integrating environmental, animal, and human health research in a One Health approach.

## Methods

### Isolation of *Pigmentiphaga litoralis* and characterisation of pPLE30.2

*P. litoralis* E30 was isolated from freshwater sediment and cultured at ambient temperature on R2A agar supplemented with 0.5 mM cadmium acetate. Sediment was obtained from the top 50 mm layer of heavy metal contaminated sediment at Crooked Creek, adjacent to the Eraring Power Station coal ash settling basin in Lake Macquarie, NSW, Australia (33°3'41” S, 151°32'52” E) in May 2021.

*P. litoralis* DNA was extracted using a cetyltrimethylammonium bromide method^57^. Stationary phase cells were suspended (10 mM Tris-HCl, 1 mM ethylenediaminetetraacetic acid, 1 M NaCl, pH 8) and incubated with lysozyme (1 mg/mL) and RNAse A (300 µg/mL) for 1 h at 37 °C. Cells were lysed with 10 mg/mL cetyltrimethylammonium bromide and proteins digested using 0.5 mg/mL proteinase K for 1 h at 70 °C with intermittent inversions. An equal volume of chloroform/isoamyl alcohol (24:1 v/v) was added, mixed by inversion, and incubated on ice for 30 min. After cell debris was removed (10,000 *g*, 4 °C, 30 min), the DNA was purified through the addition of an equal volume of phenol/chloroform/isoamyl alcohol (25:24:1 v/v/v), mixing by inversion, and centrifugation (20,000 *g*, 5 min). The recovered aqueous phase was further purified with an equal volume of chloroform/isoamyl alcohol (24:1 v/v) and centrifuged (20,000 *g*, 5 min). DNA was precipitated in 70% (v/v) ethanol and 100 mM sodium acetate for 2 h at -30 °C. DNA was collected by centrifugation (15,000 *g*, 4 °C, 30 min), washed (80% v/v ethanol, 10 mM Tris-HCl, pH 8), centrifuged again (15,000 *g*, 15 min), dried (60 °C, 15 min), and resuspended (10 mM Tris-HCl, 1 mM ethylenediaminetetraacetic acid, pH 8).

The complete genomes of two *P. litoralis* colonies were sequenced by Oxford nanopore on R10.4.1 flow cells with basecalling by Guppy v6.5.7. Returned FASTQ reads above 1 kb in length were retained using Filtlong v0.2.1^58^ and quality was assessed using Nanoplot v1.36.2^59^. Contigs were assembled with Flye v2.9.3 using automatic minimum overlap between reads, metagenomic assembly, and one polishing iteration^60^. The 16S rRNA gene was identified by barrnap^61^ and aligned with the NCBI core nucleotide database, limited to sequences from the type-strain.

Both assembled sequences contained pPLE30.2, with 57 and 72 x coverage, respectively. This high sequencing depth, and recent improvements in the accuracy of Oxford Nanopore sequencing^62^, produced 40 single or dinucleotide polymorphisms, which were reconciled depending on whether they occurred in coding or non-coding regions. For coding regions, the nucleotide that was most consistent with codon usage for *P. litoralis* was retained. Codon usage was calculated by quantifying codon proportions across all open reading frames. All nucleotide polymorphisms occurring outside coding regions occurred within mononuclear repeats ( > 4 Ns); in these cases, the longer repeat was retained, as Oxford Nanopore with Guppy base calling tends to delete bases in homopolymeric regions^63^. This reconciled genome sequence is deposited in BioProject PRJNA1263914.

The open reading frames of the plasmid were predicted by Prokka v1.14.6^64^ and annotated by applying a BLASTP alignment threshold of amino acid identity > 30%, similarity > 50%, query coverage > 50%, and *E*-value < 1 × 10^-20^. Specific gene names were assigned if amino acid identity exceeded 70%. Antibiotic resistance genes were annotated using the CARD v3.2.4^65^ and AMRFinder database v3.11.4^66^, and metal resistance genes were annotated using BacMet v2.0^67^.

DNA alignments to the plasmid backbone were identified by submitting 500 bp increments to BLASTN. Insertion sequences were identified with ISFinder^68^ with an *E*-value threshold < 1 × 10^-20^. Prophage sequences were identified using PHASTEST v1.0.0^69^. The plasmid type was determined with PlasmidFinder v2.1^70^, MOBScan^71^, and MOB-suite v3.1.8^72^.

Plasmid pPLE30.2 was cured by overnight incubation at 37 °C in 40 µg/mL acridine orange. Plasmid curing was confirmed by PCR of plasmid-specific amplicon targets (Supplementary Table 4). The MIC was measured as OD_600_ after 48 h of growth at 30 °C, 200 rpm after inoculating plasmid-carrying and plasmid-cured *P. litoralis* to an initial OD_600_ of 0.01 in a 48-well polystyrene plate containing 500 µL of cationic-adjusted Mueller-Hinton broth supplemented with serial dilutions of polymyxin B sulphate, ampicillin sodium salt, carbenicillin disodium salt, piperacillin sodium salt, zinc sulphate, cobalt chloride, cadmium acetate, sodium arsenate, sodium arsenite, and copper sulphate. Sensitivities to cephalothin, ceftazidime, cefotaxime, ceftriaxone, cefepime, meropenem, and imipenem Oxoid antimicrobial susceptibility discs (ThermoFisher Scientific) were assessed using the modified Stokes’ method^73^. Briefly, a four mm depth cation-adjusted Mueller-Hinton agar plate was divided into three segments. Early exponential phase cells (0.125 < OD_600_ < 0.25) of plasmid-carrying *P. litoralis* was applied evenly by a swab to the centre segment, while the plasmid-cured *P. litoralis* was inoculated to the two exterior segments. Four evenly spaced antibiotic discs were placed along the boundary between strains within 15 min of inoculation. Plates were incubated at 30 °C for 48 h. Annular radius were measured, where a > 3 mm paired difference in inhibition zones for an antibiotic represented a change in resistance^73^. A change in resistance in this study refers to its microbiological definition as the acquisition of a specific genetic resistance mechanism impacting a susceptibility phenotype^74^.

Mobilisation of pPLE30.2 was tested with the biparental conjugation of *P. litoralis* (pPLE30.2) donor and rifampicin-resistant, plasmid-cured *P. litoralis* receptor, while triparental conjugation was tested by adding *E. coli* HB101 (pRK2013)^75^. Mid-exponential growth phase cultures were mixed in ratios ranging from 1:1:1 through to 1:10:2 for donor/recipient/helper cells and incubated overnight on modified tryptic soy broth agar (MTSB: 20 g/L bacteriological peptone, 2.2 g/L glucose, 5 g/L NaCl, 10 mM MOPS, pH 7) at 16, 25, or 30 °C. Transconjugants were selected on MTSB with 20 µg/mL rifampicin and either 1 mM cadmium acetate or 2 mM sodium arsenate.

### Structural analysis of MCR-12

The MUSCLE algorithm in MEGA11^76^ was used to align the MCR-12 sequence to proteins from the SwissProt database or CARD with a homology *E*-value < 1 × 10^-6^. A phylogenetic maximum likelihood tree was constructed using the complete aligned protein sequences with 100 bootstrap replications. Structural models of the protein were produced using SWISS-MODEL with automodel parameters^77^, and Colabfold v1.5.5: AlphaFold2 using MMSeq2^78^. The AlphaFold structure was assembled without template information, MSA mode: mmseqs2 uniref env, pair mode: paired unpaired, model type: auto with 200 relax max iterations and greedy pairing strategy. Secondary structures were predicted using ESPript v3.0^79^.

### Cloning of *mcr-12*, *mcr-1*, and *bla*_OXA-1383_

The *mcr-12* gene was amplified by PCR (Supplementary Table 4) and ligated into vectors pBBR1MCS-2, pBBR1MCS-5^20^, or pVLT33^80^ to assemble pBBR1MCS-2-*mcr-12*, pBBR1MCS-5-*mcr-12*, and pVLT33-*mcr-12*, respectively. The *mcr-1* sequence (GenBank: AKF16168.1) was supplied as a gBlock (Integrated DNA Technologies, Singapore) and cloned using the same methodology as *mcr-12*. These construct sequences were verified by Sanger sequencing and were then electroporated into electrocompetent strains listed in Supplementary Table 5.

The fitness cost of maintenance of the plasmid and expression of *mcr-12* was assessed by comparisons of the reduction of tetramethyl-*p*-phenylenediamine dihydrochloride dye (Biolog Redox Dye, Biolog 74221), a measure of cellular respiration that is proportional to cell growth^81^. Maintenance and expression were determined to have a negligible fitness cost by comparing wild-type cells with those containing the vector and the vector with *mcr-12*. An exception for this was *P. aeruginosa* PAO1, *P. aeruginosa* PA14, and *A. baumannii* ATCC 17978, where expression of *mcr-12* marginally slowed cell growth during the exponential growth phase, though it reached the same cell density at the stationary growth phase (Supplementary Fig. 6).

The *bla*_OXA-1383_ gene was cloned into pBBR1MCS-2 and electroporated into *E. coli* TOP10. The susceptibility of *E. coli* transformed with *bla*_OXA-1383_ was assessed by MIC or modified Stokes’ method as for the β-lactams listed in Supplementary Table 3, with incubations for 18 h at 37 °C.

### Minimum inhibitory concentration

MICs towards polymyxin B or colistin (as sulphate salts) were assessed by the reduction of tetramethyl-*p*-phenylenediamine dihydrochloride dye. Mid-exponential growth phase cultures (0.4 < OD_600_ < 0.8) were inoculated into flat-bottomed polystyrene 96-well plates at approximately 5 × 10^4^ cells per well. Wells contained doubling concentrations of polymyxin B or colistin (stock prepared daily) and 1 X Biolog Redox Dye in 100 µL cation-adjusted Mueller-Hinton broth. Isopropyl ß-D-1-thiogalactopyranoside (0.25 mM) was included in the growth cultures and well plates for all *A. baumannii* strains. The absorbance at 590 nm was measured on an Odin L System (Biolog) for 96 h at 30 °C for *P. litoralis*, 48 h at 30 °C for *P. protegens*, and 48 h at 37 °C for all other strains. An MIC threshold was set as OD_590_ < 0.1.

### RT-qPCR of polymyxin resistance genes

RT-qPCR was applied to examine the expression of *mcr-12* in *P. litoralis* in the presence of polymyxin B, and whether the expression of *E. coli* endogenous PEtN transferase genes (*pmrC*, *eptB*, *eptC*) and an L-Ara4N transferase gene (*arnT*) were affected by the expression of *mcr-12*. RNA was extracted from exponential phase *P. litoralis* grown in the presence of 0, 0.02, 0.2, and 2 µg/mL polymyxin B, or from exponential and stationary phase *E. coli* (pBBR1MCS-2) or (pBBR1MCS-2-*mcr-12*) using the miRNeasy Mini Kit (217004, Qiagen) without the optional DNase digest. DNA was instead removed in the eluted RNA by following the Turbo DNA-*free*^TM^ Kit protocol (AM1907, ThermoFisher Scientific). cDNA was prepared using the Superscript^TM^ IV First-Strand Synthesis System (18091050, ThermoFisher Scientific) with random hexamer primer and without RNase H treatment.

For testing whether *mcr-12* is induced, RT-qPCR for each transcript was performed using 1 X iTaq Universal SYBR Green Supermix (Bio-Rad, 1725121) and 500 µM primers (Supplementary Table 4) on a LightCycler® 480 Instrument II. Levels of each transcript were examined in cDNA extracts (*n* = 3) using *repB* and *rpoB* as plasmid and chromosomal housekeeping control genes, respectively. Significance of differences (α = 0.05) was calculated using an ANOVA or Kruskal-Wallis test with normality and equality of variance assumptions assessed by a Shapiro-Wilk test and Levene’s test, respectively.

RT-qPCR for endogenous PEtN and L-Ara4N transferase genes were performed similarly in 1 X SensiFAST^TM^ SYBR® No-ROX Kit (Bioline, BIO-98005) on a QuantStudio^TM^ 3 Real Time PCR System, 96-well, 0.2 mL using the Design and Analysis Software v2.8.0 comparative-Ct-SYBR fast protocol with melt curve (ThermoFisher Scientific). *rpoB* was used as the housekeeping control gene and significance of differences was calculated using a one-tailed t-test with variance determined by an F-test.

### Lipid A structural analysis

Extraction of lipid A was performed according to Leung, et al.^82^ with minor modification. In brief, overnight 5 mL cultures of *P. litoralis* plasmid-carrying or plasmid-cured, and *E. coli* TOP10 or *P. protegens* Pf-5 with pBBR1MCS-2 or pBBR1MCS-2*-mcr-12* were centrifuged (7000 *g*, 5 min) and treated with 400 µL of 70% (v/v) isobutyric acid/1 M ammonium hydroxide at 100 °C for 45 min. The lysed contents were centrifuged (2000 *g*, 15 min), and the supernatant was transferred to an equivalent volume of ultrapure water, and then dried overnight at ambient conditions in a rotary evaporator. The dried contents were washed twice in methanol prior to resuspension in 50–200 µL of 2 parts chloroform:1 part methanol: 0.25 parts water.

Aliquots of extracted lipid A (*n* = 2 for *E. coli* and *P. protegens*, *n* = 3 for *P. litoralis*) mixed with matrix norhamane were analysed using an UltrafleXtreme MALDI-TOF/TOF mass spectrometer (Bruker Daltonics Inc.), operated using flexControl (v3.4, Bruker Daltonics Inc.) in negative reflection mode. Analyses were performed at 40% global intensity, and mass spectra were acquired from the sum of 5000 laser shots from 5 separate positions using the following parameter set: ion source 1: 20 kV; ion source 2: 17.8 kV; lens: 6.8 kV; reflector 1: 21.1 kV; reflector 2: 10.8 kV; pulsed ion extraction: 70 ns and detection gain: 7.7. Raw spectra were annotated in flexAnalyst (v3.4, Bruker Daltonics Inc.), following application of an external calibration obtained from analysis of a peptide calibrant mix (Bruker Daltonics Inc.) that had been spotted on a neighbouring target and analysed with the same instrument settings.

### Reporting summary

Further information on research design is available in the Nature Portfolio Reporting Summary linked to this article.

## Supplementary information

Supplementary Information

Reporting Summary

Transparent Peer Review file

## Source data

Source Data

## Data Availability

FASTA files for the *P. litoralis* E30 chromosome and plasmid pPLE30.2 were uploaded to GenBank and are publicly available under accession numbers CP184479 and PQ035968, respectively. Raw sequence reads are available under SRA accession PRJNA1223691. PDB files for ColabFold protein models and mzXML files for lipid A Mass Spectrometry are available on FigShare: Novel polymyxin resistance gene family *mcr-12* from environmental *Pigmentiphaga litoralis* [10.6084/m9.figshare.c.8326865]. All plasmids, primers, and gblocks mentioned are available for reuse by contacting the corresponding author. The publicly available datasets used in this study are listed in the Methods. Source data are provided with this paper.
