# Supplementary material for: Novel polymyxin resistance gene family mcr-12 from environmental Pigmentiphaga litoralis
Source: Nat Commun. 2026 Jul 15;17:6187. doi: 10.1038/s41467-026-75587-4 (PMC13373230; doi:10.1038/s41467-026-75587-4)
Supplement: Supplementary file 1 — Supplementary Information [file 41467_2026_75587_MOESM1_ESM.pdf]

Supplementary Information

Novel polymyxin resistance gene family *mcr-12* from environmental *Pigmentiphaga litoralis*

Supplementary Figures……………………………………………………………………………….………2

Supplementary Tables……………………………………………………………………………..………8

Supplementary References…………………………………………………………………………….13

Supplementary Figures

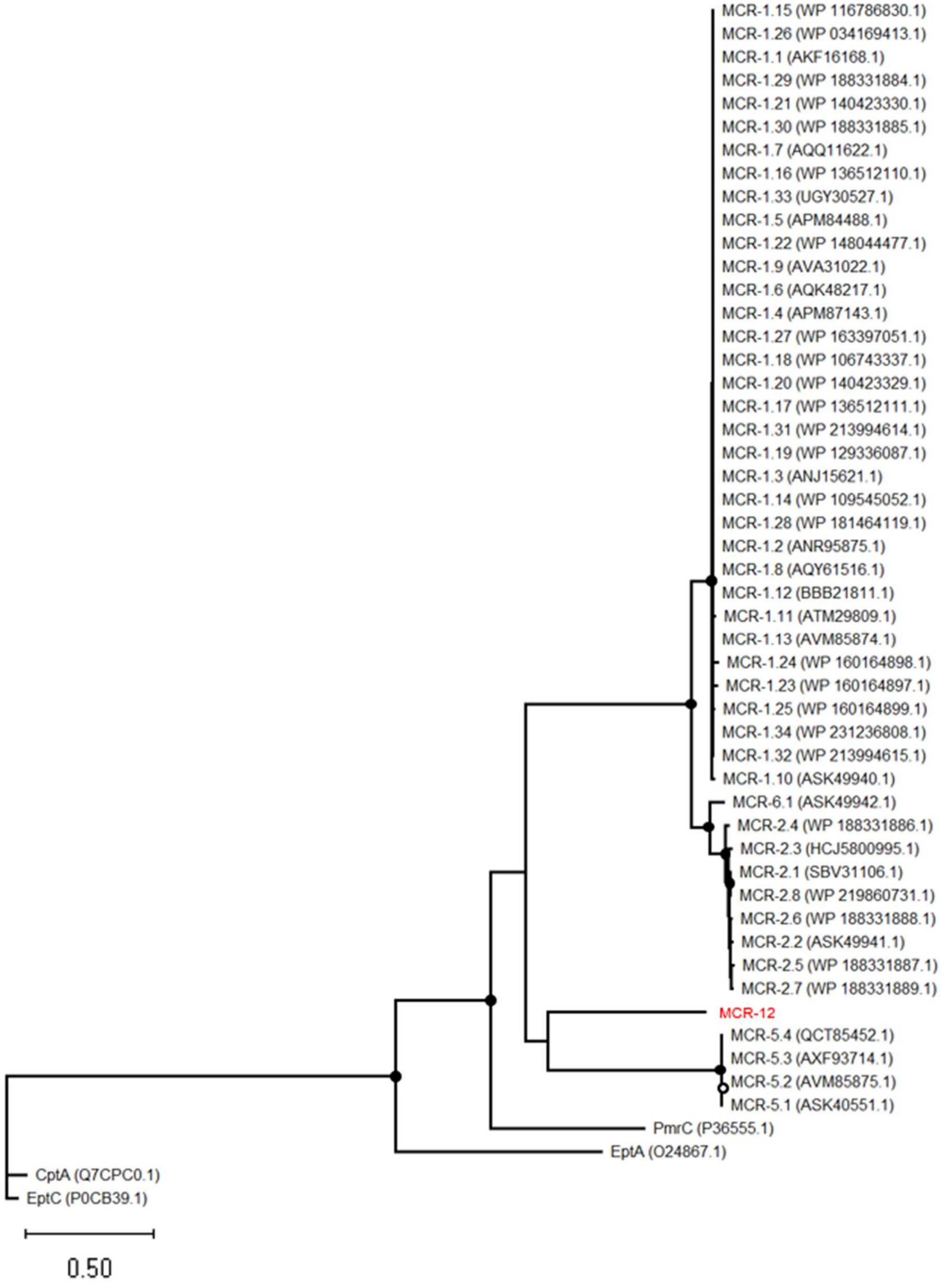

Supplementary Fig. 1: Unrooted maximum likelihood phylogeny tree of MCR-12, MCR-1, MCR-2, MCR-5, and MCR-6 families. Protein entries also include SwissProt entry alignments with an identity to MCR-12 (in red font) with *E*-value < $1x10^{-6}$. Open circle indicates > 70% bootstrap support, filled circles indicate > 85% bootstrap support. Source data are provided as a Source Data file.

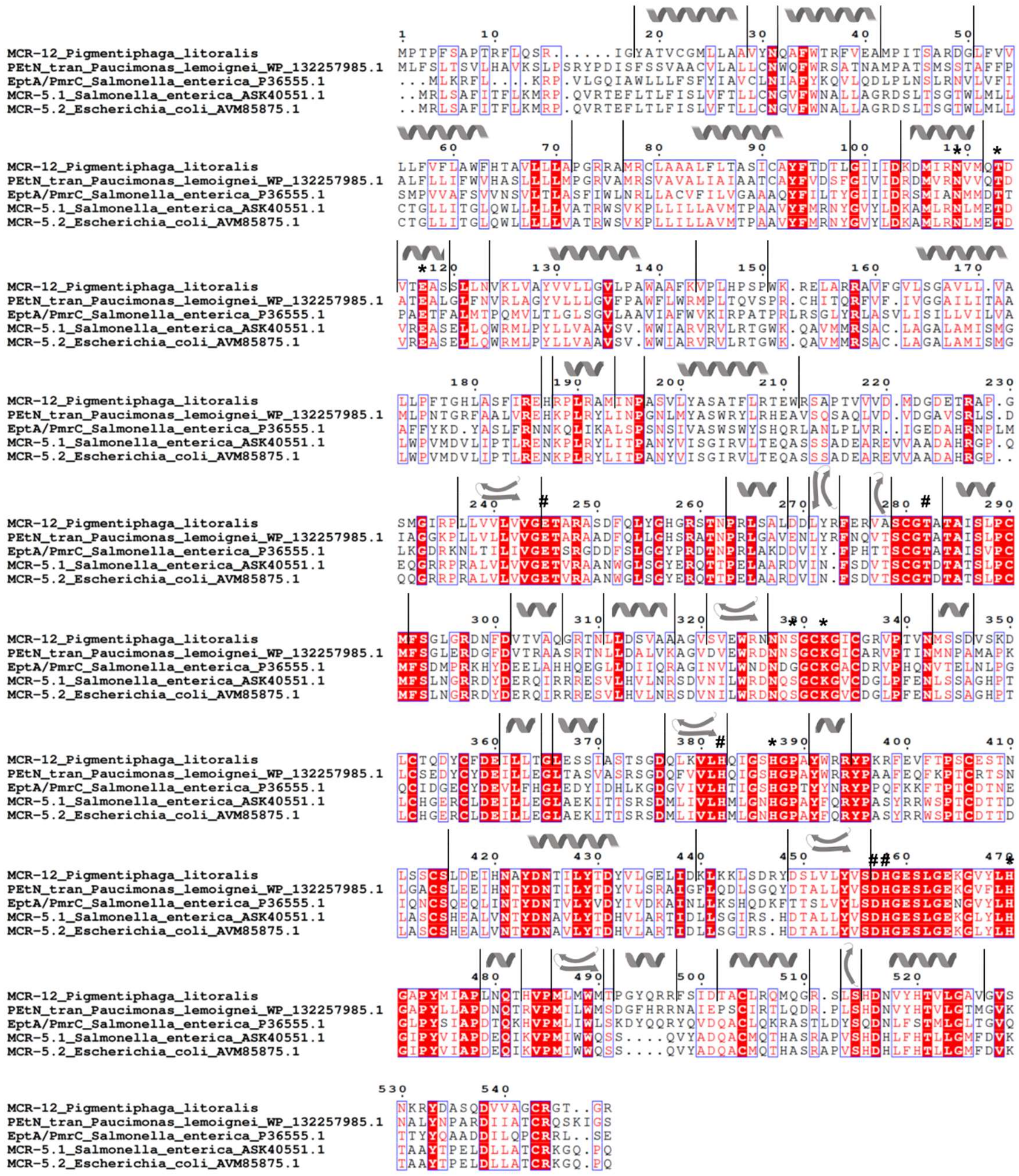

Supplementary Fig. 2: Protein sequence alignment of MCR-12 with closest homologues, MCR-5.1, MCR-5.2, EptA/PmrC, and an uncharacterised phosphoethanolamine transferase (PEtN tran). GenBank accession number listed with protein name. Identical amino acids highlighted in red by ESPript 3.0[1]. Asterix (*) indicates amino acid residues for phosphatidylethanolamine entry, and hash (#) indicates zinc binding residues. Alpha helix and beta sheet structures indicated by respective cartoon structure icon demarcated by straight lines; icons created in BioRender. Cain, A. (2026) https://BioRender.com/gda7rgz.

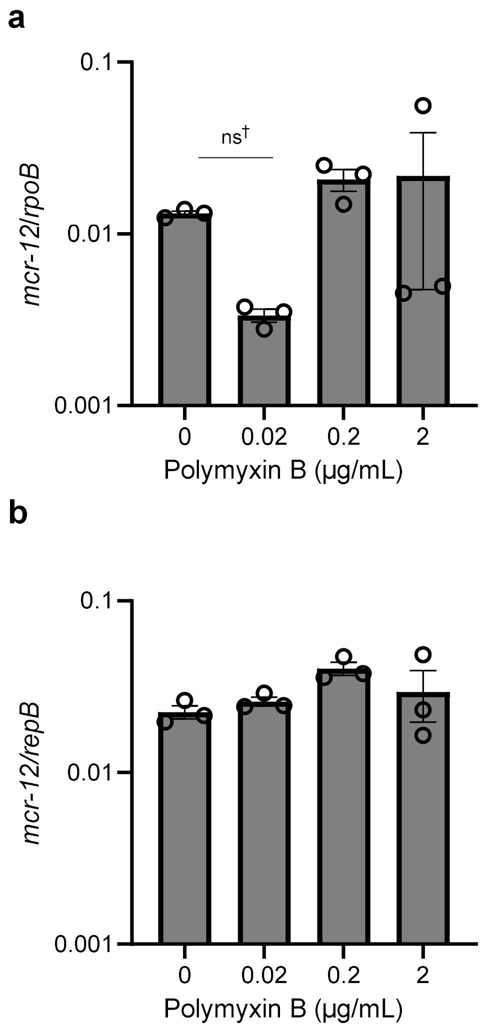

Supplementary Fig. 3: Relative expression of *mcr-12* normalised to (a) chromosomal housekeeping gene, *rpoB*, and (b) plasmid housekeeping gene, *repB*. RNA was extracted from exponential phase *Pigmentiphaga litoralis* grown in the presence of serial dilutions of polymyxin B. RNA was converted to cDNA and relative transcript abundances of *mcr-12* were measured using reverse transcription-quantitative PCR. Replicate values (open circles) displayed with mean (bar) ± standard error of mean (*n* = 3, each consisting of 3 technical replicates) on a $\log_{10}$ scale. † All differences were not significant as calculated by a (**a**) Kruskal-Wallis test and (**b**) ANOVA test. Source data are provided as a Source Data file.

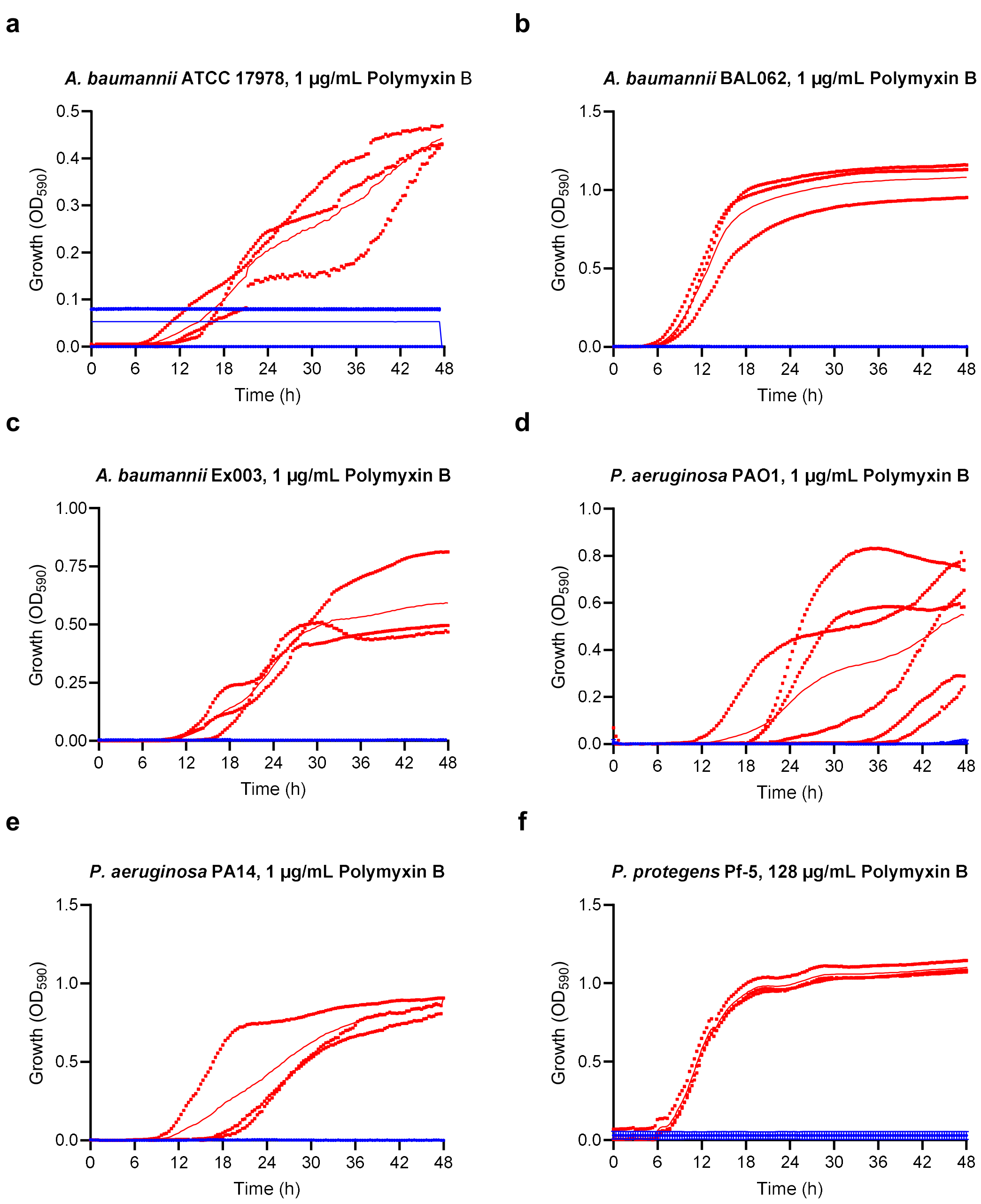

Supplementary Fig. 4: Cells with *mcr-12* were resistant to polymyxin B. Growth time course of strains transformed with *mcr-12* (red square), or a vector control (blue circle), exposed to 1 µg/mL polymyxin B (unless indicated otherwise) and measured using Biolog's Odin L System for 48 hours: (**a**) *Acinetobacter baumannii* ATCC 17978, (**b**) *A. baumannii* BAL062, (**c**) *A. baumannii* Ex003, (**d**) *Pseudomonas aeruginosa* PAO1, (**e**) *P. aeruginosa* PA14, and (**f**) *Pseudomonas protegens* Pf-5 exposed to 128 µg/mL polymyxin B. Data represents independent replicates with the mean as a line (n = 3, apart from panel d where n = 6, each with 2–4 technical replicates). Source data are provided as a Source Data file.

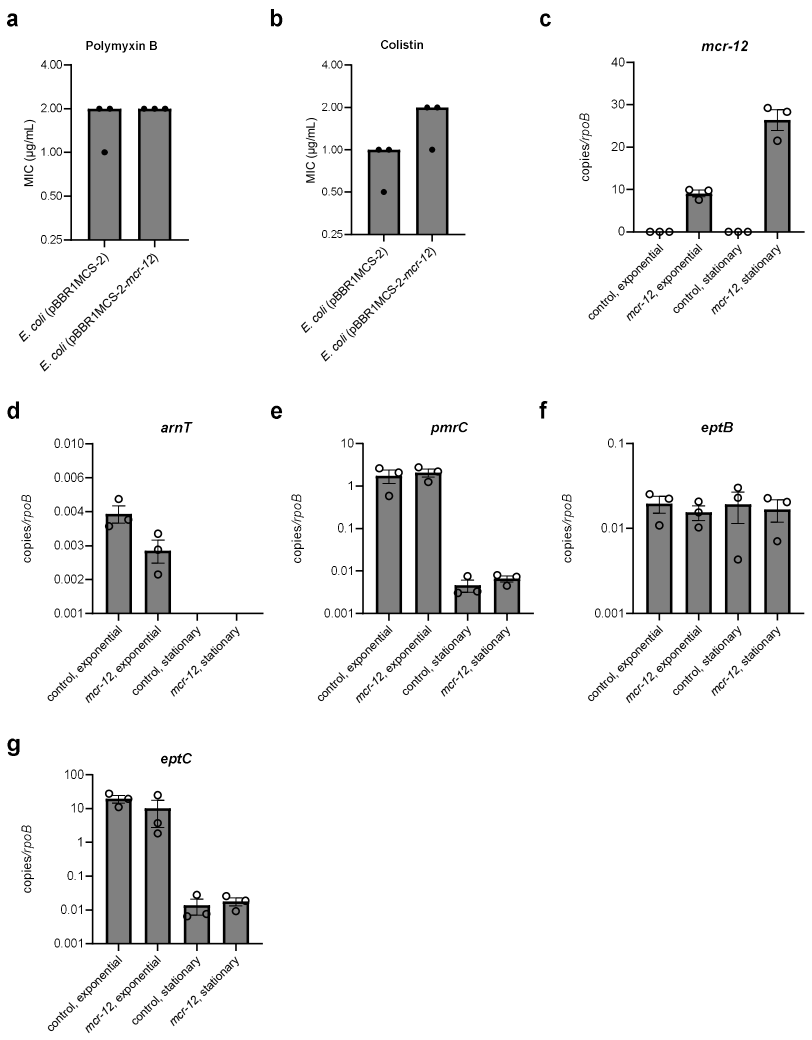

Supplementary Fig. 5: Establishing the contribution of endogenous phosphoethanolamine transferases and an endogenous 4-amino-4-deoxy-L-arabinose transferase to *mcr-12*-mediated polymyxin resistance in *Escherichia coli*. Minimum inhibitory concentrations (MICs) of polymyxin B (**a**) and colistin (**b**) for *E. coli* TOP10 expressing *mcr-12*, or transformed with a vector control, measured by OD_{600} after 48 hours. Independent replicates (*n* = 3, each with 2–4 technical replicates) displayed with the median MIC as a column. RNA was extracted from *E. coli* TOP10 cells transformed with pBBR1MCS-2 (control), or pBBR1MCS-2-*mcr-12* (mcr-12), at mid-exponential phase (OD_{600nm} = 0.5) or stationary phase and converted to cDNA. Relative transcript abundance of gene targets (**c**) *mcr-12*, (**d**) *arnT*, (**e**) *pmrC*, (**f**) *eptB*, and (**g**) *eptC* were measured using reverse transcription-quantitative PCR, normalised to the amount of *rpoB* transcript copies. Replicate values (open circles) displayed with mean (column) ± standard error of mean (*n* = 3, each with 1–3 technical replicates) on a $\log_{10}$ scale. Differences in **d**–**g** were not significant as calculated by a one-tailed t-test. Source data are provided as a Source Data file.

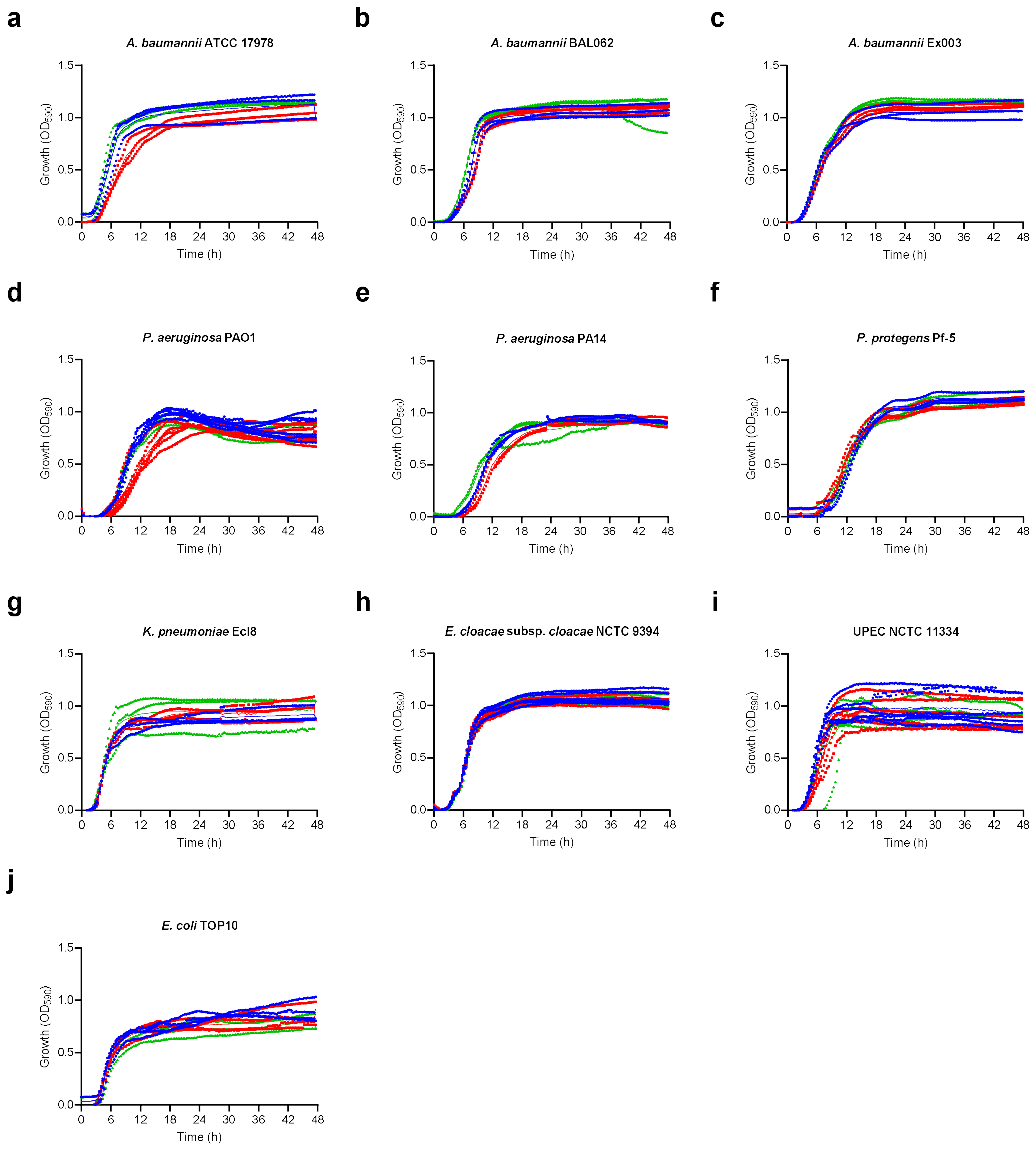

Supplementary Fig. 6: Cell growth was unaffected by *mcr-12* expression. Growth of wild-type strains (green triangle), strains expressing *mcr-12* (red square), or a vector control (blue circle) was set up following minimum inhibitory concentration inoculation conditions, but without polymyxin B, and measured using the Biolog's Odin L System. (**a**) *Acinetobacter baumannii* ATCC 17978, (**b**) *A. baumannii* BAL062, (**c**) *A. baumannii* Ex003, (**d**) *Pseudomonas aeruginosa* PAO1, (**e**) *P. aeruginosa* PA14, (**f**) *Pseudomonas protegens* Pf-5, (**g**) *Klebsiella pneumoniae* Ecl8, (**h**) *Enterobacter cloacae* subsp. *cloacae* NCTC 9394, (**i**) uropathogenic *Escherichia coli* (UPEC) NCTC 11334, (**j**) *E. coli* TOP10. Data represents independent replicates with the mean as a line (n = 3, apart for n = 2 for wild-type in panel **a** and **j**; n = 4 for wild-type in panel **h** and **i**, and vector and *mcr-12* insert in panel **j**; n = 6 for vector and *mcr-12* insert in panel **d**, **h**, and **i**; all independent replicates consists of 2–4 technical replicates). Source data are provided as a Source Data file.

Supplementary Tables

Supplementary Table 1: Discoveries of *mcr* gene families. The host bacterium, sample source, country, and year(s) in which the sample(s) originated are listed along with the mobile genetic element(s) that the *mcr* gene resided within.

***mcr* family**	**Host**	**Taxonomic family**	**Source**	**Country**	**Year**	**Mobile genetic element**	**Reference**
mcr-1	*Escherichia coli*, *Klebsiella pneumoniae*	Enterobacteriaceae	Swine, human	China	2011-14	pHNSHP45 (IncI2)	[2]
mcr-2	*E. coli*	Enterobacteriaceae	Bovine, swine	Belgium	2011-12	IS*1595*, pKP37-BE (IncX4)	[3]
mcr-3	*E. coli*	Enterobacteriaceae	Swine, Human	China, US, Malaysia	2015	Tn*As2*, pWJ1 (IncHI2)	[4]
mcr-4	*Salmonella enterica, E. coli*	Enterobacteriaceae	Swine	Italy, Spain, Belgium	2013-2016	pMCR (ColE)	[5]
mcr-5	*S. enterica*	Enterobacteriaceae	Poultry	Germany	2011-13	Tn*3*, pSE12, pSE13 (ColE)	[6]
mcr-6	*Moraxella pluranimalium*	Moraxellaceae	Swine	UK	2014-15	Non-functional IS*1595*	[7]
mcr-7	*K. pneumoniae*	Enterobacteriaceae	Poultry	China	2010-15	pSC20141012 (IncI2)	[8]
mcr-8	*K. pneumoniae*	Enterobacteriaceae	Swine, Human	China	2015-17	IS*903B*, IS*Ecl1*, pKP91 (IncFII)	[9]
mcr-9	*S. enterica*	Enterobacteriaceae	Human	US	2010	IncHI2	[10]
mcr-10	*Enterobacter roggenkampii*	Enterobacteriaceae	Human	China	2016	IS*903*, IncFIA	[11]
mcr-11	*Leclercia adecarboxylata*	Enterobacteriaceae	Not given†	China	2019	Not given†	Unpublished
mcr-12	*Pigmentiphaga litoralis*	Alcaligenaceae	Sediment	Australia	2021	pPLE30.2	This study

† Information available from GenBank accession number WP_150870284.

Supplementary Table 2: Susceptibility to β-lactams was unchanged between *Pigmentiphaga litoralis* (pPLE30.2) and pPLE30.2-cured isogenic strains. Source data are provided as a Source Data file.

β-lactam	*P. litoralis* (pPLE30.2)	*P. litoralis* (pPLE30.2⁻)
**Minimum inhibitory concentration (µg/mL)†**		
Ampicillin	>1,000	>1,000
Carbenicillin	>1,000	>1,000
Piperacillin	>1,000	>1,000
**Modified Stokes' method: zone of inhibition annular radius (mm)‡**		
Cefepime	None	None
Cefotaxime	5.2 ± 0.2	5.0 ± 0.0
Ceftazidime	10.0 ± 0.3	9.8 ± 0.2
Ceftriaxone	8.5 ± 0.5	7.5 ± 0.5
Cephalothin	None	None
Imipenem	11.2 ± 0.4	10.3 ± 0.2
Meropenem	10.8 ± 0.7	11.5 ± 0.3

† Minimum inhibitory concentration (MIC) determined by an OD_{600} < 0.1 after 48 hours growth in cation-adjusted Mueller-Hinton broth. MICs are consensus of n = 2, each with 2–3 technical replicates.

‡ Annular radii (mm) from a modified Stokes' method disc diffusion are means ± standard error of mean (n = 3). A paired difference in inhibition zones > 3 mm represents a significant difference[12].

Supplementary Table 3: β-lactam susceptibility profile of *Escherichia coli* TOP10 was unaltered when transformed with pBBR1MCS-2 or pBBR1MCS-2-*bla*$_{\text{OXA-1383}}$. Source data are provided as a Source Data file.

β-lactam	*E. coli* (pBBR1MCS-2)	*E. coli* (pBBR1MCS-2-*bla*$_{\text{OXA-1383}}$)
**Minimum inhibitory concentration (µg/mL)†**		
Ampicillin	6.3	6.3
Carbenicillin	5.0	5.0
Piperacillin	5.0	5.0
**Modified Stokes' method: zone of inhibition annular radius (mm)‡**		
Cefepime	16.7 ± 0.2	16.0 ± 0
Cefotaxime	17.0 ± 0.3	16.7± 0.2
Ceftazidime	16.2 ± 0.2	16.5 ± 0.3
Ceftriaxone	12.3 ± 0.2	12.3 ± 0.2
Cephalothin	4.0 ± 0	3.3 ± 0.3
Imipenem	14.0 ± 0	14.7 ± 0.7
Meropenem	17.2 ± 0.2	16.0 ± 0.3

† Minimum inhibitory concentration (MIC) determined by an OD_{600} < 0.1 after 18 hours growth in cation-adjusted Mueller-Hinton broth. MICs are consensus of n = 3 (ampicillin) or n = 2 (carbenicillin and piperacillin), all with 2-4 technical replicates.

‡ Susceptibility determined through a modified Stokes' method where a paired difference in inhibition zones > 3 mm represents a significant difference[12]. Annular radii are means ± standard error of mean (n = 3).

Supplementary Table 4: Primers and gBlocks used in this study. All primers and gBlocks were designed specifically for this study, apart from NVC15b, NVC16, MVS166, and LFE5 which were designed as part of a previous unpublished study. All primers and gBlocks were synthesised by Integrated DNA Technologies, Singapore.

Primer	Sequence (5' – 3')	Purpose
BFG90	AAAGGTACCATAACACACAAAGGAGGCTTTATCATGCCAACCCCG TTTTCG	Cloning of *mcr-12* from pPLE30.2
BGF91	TTTAAGCTTTGGTGAGTGAGATCTGCAGAGTTTC	
BFG88	TTAAAGCTTCGGAGCGACACTGTTCTTTC	Cloning of *bla*$_{OXA-1383}$ from pPLE30.2
BFG157	AAAGGTACCATAACACACAAAGGAGGCTGAATGTTCAAAGCCTCA GCG	
NVC15b	CTTCCGGCTCGTATGTTGTG	Spanning PCR, pBBR1MCS
NVC16	CAAGGCGATTAAGTTGGGTAACG	
MVS166	CAAGGCGATTAAGTTGGGTAACG	Spanning PCR, pVLT33
LFE5	CAAGGCGATTAAGTTGGGTAACG	
BFG145	TGAACACTTACGACAACACGCTGGTTTACG	Reverse transcription-quantitative PCR (RT-qPCR) *pmrC* (*Escherichia coli*)
BFG146	TCACCTAACGATTCACCGTGGTCAGAAAGA	
BFG147	GCGTCTGCTCTGGCAACCCG	RT-qPCR *arnT* (*E. coli*)
BFG148	ACAGATGGGGCACAATCCAGTCG	
BFG149	TCCGACAACTGCGAACCCCA	RT-qPCR *eptB* (*E. coli*)
BFG150	AGTTCGCTACGACACCGCCA	
BFG151	TCCACAGCAGGAACGGAATGGTG	RT-qPCR *eptC* (*E. coli*)
BFG152	GACACGCCGCCGCACAAAA	
BFG153	GGTATCCAGATCGCCCAGAGACAGAC	RT-qPCR *rpoB* (*E. coli*)
BFG154	ATATCGACCACCTCGGCAACCG	
BFG155	GGTCTGTTCGTCGTGCTCCTGTTC	RT-qPCR *mcr-12*
BFG156	CGATGCCGTCAGAAACAACGCC	
BFG179	CAGGGGCGATGCGAAGGTCA	RT-qPCR *rpoB* (*Pigmentiphaga litoralis*)
BFG180	TTCCCCATCGTCAGCCACAACC	
BFG184	CGGAAGCCGCAACCACAAGC	RT-qPCR *repB* (*P. litoralis*)
BFG185	TGAGCTGCACTGACGACCACTT	
gBFG3	AAAGAATTCATAACACACAAAGGAGGCTTTATCATGATGCAGCAT ACTTCTGTGTGGTACCGACGCTCGGTCAGTCCGTTTGTTCTTGTGG CGAGTGTTGCCGTTTTCTTGACCGCGACCGCCAATCTTACCTTTTTT GATAAAATCAGCCAAACCTATCCCATCGCGGACAATCTCGGCTTTG TGCTGACGATCGCTGTCGTGCTCTTTGGCGCGATGCTACTGATCAC CACGCTGTTATCATCGTATCGCTATGTGCTAAAGCCTGTGTTGATT TTGCTATTAATCATGGGCGCGGTGACCAGTTATTTTACTGACACTT ATGGCACGGTCTATGATACGACCATGCTCCAAAATGCCCTACAGA CCGACCAAGCCGAGACCAAGGATCTATTAAACGCAGCGTTTATCA TGCGTATCATTGGTTTGGGTGTGCTACCAAGTTTGCTTGTGGCTTT TGTTAAGGTGGATTATCCGACTTGGGGCAAGGGTTTGATGCGCCG	Cloning of *mcr-1*. *mcr-1* from Liu, et al. [2]

	ATTGGGCTTGATCGTGGCAAGTCTTGCGCTGATTTTACTGCCTGTG<br>GTGGCGTTCAGCAGTCATTATGCCAGTTTCTTTCGCGTGCATAAGC<br>CGCTGCGTAGCTATGTCAATCCGATCATGCCAATCTACTCGGTGG<br>GTAAGCTTGCCAGTATTGAGTATAAAAAAGCCAGTGCGCCAAAAG<br>ATACCATTTATCACGCCAAAGACGCGGTACAAGCAACCAAGCCTG<br>ATATGCGTAAGCCACGCCTAGTGGTGTTCGTCGTCGGTGAGACGG<br>CACGCGCCGATCATGTCAGCTTCAATGGCTATGAGCGCGATACTTT<br>CCCACAGCTTGCCAAGATCGATGGCGTGACCAATTTTAGCAATGTC<br>ACATCGTGCGGCACATCGACGGCGTATTCTGTGCCGTGTATGTTC<br>AGCTATCTGGGCGCGGATGAGTATGATGTCGATACCGCCAAATAC<br>CAAGAAAATGTGCTGGATACGCTGGATCGCTTGGGCGTAAGTATC<br>TTGTGGCGTGATAATAATTCGGACTCAAAAGGCGTGATGGATAAG<br>CTGCCAAAAGCGCAATTTGCCGATTATAAATCCGCGACCAACAAC<br>GCCATCTGCAACACCAATCCTTATAACGAATGCCGCGATGTCGGTA<br>TGCTCGTTGGCTTAGATGACTTTGTCGCTGCCAATAACGGCAAAG<br>ATATGCTGATCATGCTGCACCAAATGGGCAATCACGGGCCTGCGT<br>ATTTTAAGCGATATGATGAAAAGTTTGCCAAATTCACGCCAGTGTG<br>TGAAGGTAATGAGCTTGCCAAGTGCGAACATCAGTCCTTGATCAA<br>TGCTTATGACAATGCCTTGCTTGCCACCGATGATTTCATCGCTCAA<br>AGTATCCAGTGGCTGCAGACGCACAGCAATGCCTATGATGTCTCA<br>ATGCTGTATGTCAGCGATCATGGCGAAAGTCTGGGTGAGAACGG<br>TGTCTATCTACATGGTATGCCAAATGCCTTTGCACCAAAAGAACAG<br>CGCAGTGTGCCTGCATTTTTCTGGACGGATAAGCAAACTGGCATC<br>ACGCCAATGGCAACCGATACCGTCCTGACCCATGACGCGATCACG<br>CCGACATTATTAAAGCTGTTTGATGTCACCGCGGACAAAGTCAAA<br>GACCGCACCGCATTCATCCGCTGAACCAGACCCGAAACTCTGCAG<br>ATCTCACTCACCAGGATCCAAA	

Supplementary Table 5: The heterologous expression or reintroduction of *mcr-12*. Strains were transformed by electroporation with *mcr-12* cloned into the listed plasmids, or with empty vector controls. Transformed cells were recovered for one hour at the listed temperature and selected on cation-adjusted Mueller-Hinton broth agar supplemented with the listed antibiotic concentration.

Strain	Transformed plasmid	Recovery temperature (°C)	Selection for transformed cells	Selective concentration (µg/mL)
Pigmentiphaga litoralis (pPLE30.2⁻)[†]	pBBR1MCS-2	30	Kanamycin	50
Pseudomonas aeruginosa PAO1	pBBR1MCS-5	37	Gentamicin	100
Pseudomonas aeruginosa PA14	pBBR1MCS-2	37	Kanamycin	250
Pseudomonas protegens Pf-5	pBBR1MCS-2	30	Kanamycin	50
Klebsiella pneumoniae Ecl8	pBBR1MCS-2	37	Kanamycin	50
Acinetobacter baumannii ATCC 17978	pVLT33[‡]	37	Kanamycin	50
Acinetobacter baumannii BAL062	pVLT33[‡]	37	Kanamycin	50
Acinetobacter baumannii Ex003	pVLT33[‡]	37	Kanamycin	10
Uropathogenic *Escherichia coli* NCTC 11334	pBBR1MCS-5	37	Gentamicin	100
Enterobacter cloacae subsp. *cloacae* NCTC 9394	pBBR1MCS-5	37	Gentamicin	10
Escherichia coli TOP10	pBBR1MCS-2	37	Kanamycin	50

[†] The original *P. litoralis* strain carrying pPLE30.2-borne *mcr-12* was cured of its plasmid, forming the plasmid-cured strain.

[‡] Used instead of pBBR1MCS as these do not replicate in *Acinetobacter*.

Supplementary References

1 Robert, X. & Gouet, P. Deciphering key features in protein structures with the new ENDscript server. *Nucleic Acids Res* **42**, W320-W324 (2014).

2 Liu, Y.-Y. *et al.* Emergence of plasmid-mediated colistin resistance mechanism MCR-1 in animals and human beings in China: a microbiological and molecular biological study. *Lancet Infect Dis* **16**, 161-168 (2016).

3 Xavier, B. B. *et al.* Identification of a novel plasmid-mediated colistin-resistance gene, *mcr-2*, in *Escherichia coli*, Belgium, June 2016. *Euro Surveill* **21**, 30280 (2016).

4 Yin, W. *et al.* Novel plasmid-mediated colistin resistance gene *mcr-3* in *Escherichia coli*. *mBio* **8**, e00543-00517 (2017).

5 Carattoli, A. *et al.* Novel plasmid-mediated colistin resistance *mcr-4* gene in *Salmonella* and *Escherichia coli*, Italy 2013, Spain and Belgium, 2015 to 2016. *Euro Surveill* **22**, 30589 (2017).

6 Borowiak, M. *et al.* Identification of a novel transposon-associated phosphoethanolamine transferase gene, *mcr-5*, conferring colistin resistance in *d*-tartrate fermenting *Salmonella enterica* subsp. *enterica* serovar Paratyphi B. *J Antimicrob Chemother* **72**, 3317-3324 (2017).

7 AbuOun, M. *et al. mcr-1* and *mcr-2* variant genes identified in *Moraxella* species isolated from pigs in Great Britain from 2014 to 2015. *J Antimicrob Chemother* **72**, 2745-2749 (2017).

8 Yang, Y.-Q., Li, Y.-X., Lei, C.-W., Zhang, A.-Y. & Wang, H.-N. Novel plasmid-mediated colistin resistance gene *mcr-7.1* in *Klebsiella pneumoniae*. *J Antimicrob Chemother* **73**, 1791-1795 (2018).

9 Wang, X. *et al.* Emergence of a novel mobile colistin resistance gene, *mcr-8*, in NDM-producing *Klebsiella pneumoniae*. *Emerg Microbes Infect* **7**, 122 (2018).

10 Carroll, L. M. *et al.* Identification of novel mobilized colistin resistance gene *mcr-9* in a multidrug-resistant, colistin-susceptible *Salmonella enterica* serotype Typhimurium isolate. *mBio* **10**, e00853-00819 (2019).

11 Wang, C. *et al.* Identification of novel mobile colistin resistance gene *mcr-10*. *Emerg Microbes Infect* **9**, 508-516 (2020).

12 Gosden, P. E. *et al.* Comparison of the modified Stokes' method of susceptibility testing with results obtained using MIC methods and British Society of Antimicrobial Chemotherapy breakpoints. *J Antimicrob Chemother* **42**, 161-169 (1998).
