# Supplementary material for: Novel polymyxin resistance gene family mcr-12 from environmental Pigmentiphaga litoralis
Source: Nat Commun. 2026 Jul 15;17:6187. doi: 10.1038/s41467-026-75587-4 (PMC13373230; doi:10.1038/s41467-026-75587-4)
Supplement: Supplementary file 2 — Reporting Summary [file 41467_2026_75587_MOESM2_ESM.pdf]

nature portfolio

Corresponding author(s): Amy K Cain
Last updated by author(s): 2026/06/17

Reporting Summary

Nature Portfolio wishes to improve the reproducibility of the work that we publish. This form provides structure for consistency and transparency in reporting. For further information on Nature Portfolio policies, see our Editorial Policies and the Editorial Policy Checklist.

Statistics

For all statistical analyses, confirm that the following items are present in the figure legend, table legend, main text, or Methods section.

n/a	Confirmed	
☐	☒	The exact sample size (*n*) for each experimental group/condition, given as a discrete number and unit of measurement
☐	☒	A statement on whether measurements were taken from distinct samples or whether the same sample was measured repeatedly
☐	☒	The statistical test(s) used AND whether they are one- or two-sided<br>*Only common tests should be described solely by name; describe more complex techniques in the Methods section.*
☒	☐	A description of all covariates tested
☐	☒	A description of any assumptions or corrections, such as tests of normality and adjustment for multiple comparisons
☐	☒	A full description of the statistical parameters including central tendency (e.g. means) or other basic estimates (e.g. regression coefficient) AND variation (e.g. standard deviation) or associated estimates of uncertainty (e.g. confidence intervals)
☐	☒	For null hypothesis testing, the test statistic (e.g. *F*, *t*, *r*) with confidence intervals, effect sizes, degrees of freedom and *P* value noted<br>*Give P values as exact values whenever suitable.*
☒	☐	For Bayesian analysis, information on the choice of priors and Markov chain Monte Carlo settings
☒	☐	For hierarchical and complex designs, identification of the appropriate level for tests and full reporting of outcomes
☒	☐	Estimates of effect sizes (e.g. Cohen's *d*, Pearson's *r*), indicating how they were calculated

Our web collection on statistics for biologists contains articles on many of the points above.

Software and code

Policy information about availability of computer code

Data collection	Data was collected as outlined in the manuscript<br>Long-read whole genome sequencing was performed by Nanopore R10.4.1 flow cells and MinION Mk1B device with basecalling by Guppy v6.5.7 at SeqCenter (USA)<br>MIC data was measured as OD590 on Biolog's Odin L System<br>Modified Stokes' method disc diffusion was measured as annular radius<br>RT-qPCR Ct values for recombinant E. coli measured by QuantStudioTM 3 Real Time PCR System. For Pigmentiphaga, Roche LigthCycler 480 II was used instead.<br>Mass-spectrometry data was collected by UltrafleXtreme MALDI-TOF/TOF mass spectrometer (Bruker Daltonics Inc.), operated in negative reflection mode
Data analysis	WGS- FASTQ files were filtered by Filtlong v0.2.1, assessed with nanoplot v1.36.2, and assembled using Flye v2.9.3 using automatic minimum overlap between read, metagenomic assembly, and one polishing iteration.<br>Gene annotation - Open readings frame were predicted by Prokka v1.14.6. and annotated by applying a BLASTP alignment threshold of amino acid identity > 30%, similarity > 50%, query coverage > 50%, and E-value < 1x10^-20. Specific gene names were assigned if amino acid identity and query exceeded 70%. Antibiotic resistance genes were annotated using the CARD v3.2.4 and AMRFinder database v3.11.4, and metal resistance genes were annotated using BacMet v2.0. DNA alignments to the plasmid backbone were identified by submitting 500 bp increments to BLASTN. Insertion sequences were identified with ISFinder with an E-value threshold < 1x10^-20. Prophage sequences were identified using PHASTEST v1.0.0. The plasmid type was determined with PlasmidFinder v2.1, MOBScan, and MOB-suite v3.1.8.<br>Phylogenetic trees - in MEGA11, protein alignments were made by MUSCLE, then maximum likelihood trees were assembled using the complete aligned protein sequence and 100 bootstrap replications.

Protein models - structural models of the protein were produced using SWISS-MODEL with automodel parameters, and Colabfold v1.5.5: AlphaFold2 using MMSeq2. The AlphaFold structure was assembled without template information, MSA mode: mmseqs2 uniref env, pair mode: paired unpaired, model type: auto with 200 relax max iterations and greedy pairing strategy. Secondary structures were predicted using ESPript v3.0.
MIC- defined as OD590 or OD600 < 0.1 at the end timepoint
Modified Stokes' method- difference in susceptibility defined as an annular radii difference >3 mm
RT-qPCR - Ct value and melt curve analysed using Design and Analysis Software v2.8.0
Mass spectrometry - Analyses were performed at 40% global intensity, and mass spectra were acquired from the sum of 5,000 laser shots from 5 separate positions using the following parameter set: Ion source 1: 20 kV; ion source 2: 17.8 kV; lens: 6.8 kV; reflector 1: 21.1 kV; reflector 2: 10.8 kV; pulsed ion extraction: 70 ns and detection gain: 7.7.

For manuscripts utilizing custom algorithms or software that are central to the research but not yet described in published literature, software must be made available to editors and reviewers. We strongly encourage code deposition in a community repository (e.g. GitHub). See the Nature Portfolio guidelines for submitting code & software for further information.

Data

Policy information about availability of data

All manuscripts must include a data availability statement. This statement should provide the following information, where applicable:

- Accession codes, unique identifiers, or web links for publicly available datasets
- A description of any restrictions on data availability
- For clinical datasets or third party data, please ensure that the statement adheres to our policy

FASTA files for the P. litoralis E30 chromosome and plasmid pPLE30.2 were uploaded to GenBank and are publicly available under accession number CP184479 [https://www.ncbi.nlm.nih.gov/nuccore/CP184479]and PQ035968 [https://www.ncbi.nlm.nih.gov/nuccore/PQ035968], respectively. Raw sequence reads are available under SRA accession PRJNA1223691 [https://www.ncbi.nlm.nih.gov/bioproject/PRJNA1223691/]. PDB files for ColabFold protein models and mzXML files for lipid A Mass Spectrometry are available on FigShare Novel polymyxin resistance gene family mcr-12 from environmental Pigmentiphaga litoralis [https://doi.org/10.6084/m9.figshare.c.8326865]. All plasmids, primers, and gblocks mentioned are available for reuse by contacting the corresponding author: amy.cain@mq.edu.au. The publicly available datasets used in this study are listed in the Methods. All other relevant data are available in the Article and Supplementary Information, with accompanying raw data available in the Source Data File.

Research involving human participants, their data, or biological material

Policy information about studies with human participants or human data. See also policy information about sex, gender (identity/presentation), and sexual orientation and race, ethnicity and racism.

Reporting on sex and gender	*Use the terms sex (biological attribute) and gender (shaped by social and cultural circumstances) carefully in order to avoid confusing both terms. Indicate if findings apply to only one sex or gender; describe whether sex and gender were considered in study design; whether sex and/or gender was determined based on self-reporting or assigned and methods used. Provide in the source data disaggregated sex and gender data, where this information has been collected, and if consent has been obtained for sharing of individual-level data; provide overall numbers in this Reporting Summary. Please state if this information has not been collected. Report sex- and gender-based analyses where performed, justify reasons for lack of sex- and gender-based analysis.*
Reporting on race, ethnicity, or other socially relevant groupings	*Please specify the socially constructed or socially relevant categorization variable(s) used in your manuscript and explain why they were used. Please note that such variables should not be used as proxies for other socially constructed/relevant variables (for example, race or ethnicity should not be used as a proxy for socioeconomic status). Provide clear definitions of the relevant terms used, how they were provided (by the participants/respondents, the researchers, or third parties), and the method(s) used to classify people into the different categories (e.g. self-report, census or administrative data, social media data, etc.) Please provide details about how you controlled for confounding variables in your analyses.*
Population characteristics	*Describe the covariate-relevant population characteristics of the human research participants (e.g. age, genotypic information, past and current diagnosis and treatment categories). If you filled out the behavioural & social sciences study design questions and have nothing to add here, write "See above."*
Recruitment	*Describe how participants were recruited. Outline any potential self-selection bias or other biases that may be present and how these are likely to impact results.*
Ethics oversight	*Identify the organization(s) that approved the study protocol.*

Note that full information on the approval of the study protocol must also be provided in the manuscript.

Field-specific reporting

Please select the one below that is the best fit for your research. If you are not sure, read the appropriate sections before making your selection.

[x] Life sciences [ ] Behavioural & social sciences [ ] Ecological, evolutionary & environmental sciences

For a reference copy of the document with all sections, see nature.com/documents/nr-reporting-summary-flat.pdf

Life sciences study design

All studies must disclose on these points even when the disclosure is negative.

Sample size	No sample size calculations were determined for MIC, modified Stokes' method, and RT-qPCR experiments. Three independent biological replicates was chosen as this is standard for these experiments; for some MIC measurements an n=2 was acceptable if these were perfectly consistent and showed no difference between treatment groups. Error bars represent standard error of mean, as indicated in each figure or table.
Data exclusions	Initial data from ampicillin MIC experiments were excluded due to degraded antibiotic stock. This data has been replaced and updated by repeated measurements in triplicate using fresh ampicillin stock for both E. coli and P. litoralis. Penicillin G and Oxacillin MIC data has been removed for both E. coli and P. litoralis MIC data as suggested by reviewers, as these these Gram-negative bacteria have intrinsic resistance.
Replication	For MICs and RT-qPCR, data was collected from three independent biological replicates, each consisting of two-four technical replicates. Each replication gave a consistent value within 2-fold of the average value. Each MIC biological replicates was carried out on a different day, and each polymyxin B MIC replicate used an independently freshly prepared polymyxin stock. Modified Stokes' method consisted of three independent biological replicates of one technical replicate. The standard deviation for all modified Stokes' method result was within 0.8 mm, so was deemed consistent.
Randomization	Randomisation was not relevant to this study as no subjective measurements were performed.
Blinding	Blinding was not performed in the data collection or analysis phase as no subjective measurements were made

Reporting for specific materials, systems and methods

We require information from authors about some types of materials, experimental systems and methods used in many studies. Here, indicate whether each material, system or method listed is relevant to your study. If you are not sure if a list item applies to your research, read the appropriate section before selecting a response.

Materials & experimental systems

n/a	Involved in the study	
☒	☐	Antibodies
☒	☐	Eukaryotic cell lines
☒	☐	Palaeontology and archaeology
☒	☐	Animals and other organisms
☒	☐	Clinical data
☒	☐	Dual use research of concern
☒	☐	Plants

Methods

n/a	Involved in the study	
☒	☐	ChIP-seq
☒	☐	Flow cytometry
☒	☐	MRI-based neuroimaging

Plants

Seed stocks	*Report on the source of all seed stocks or other plant material used. If applicable, state the seed stock centre and catalogue number. If plant specimens were collected from the field, describe the collection location, date and sampling procedures.*
Novel plant genotypes	*Describe the methods by which all novel plant genotypes were produced. This includes those generated by transgenic approaches, gene editing, chemical/radiation-based mutagenesis and hybridization. For transgenic lines, describe the transformation method, the number of independent lines analyzed and the generation upon which experiments were performed. For gene-edited lines, describe the editor used, the endogenous sequence targeted for editing, the targeting guide RNA sequence (if applicable) and how the editor was applied.*
Authentication	*Describe any authentication procedures for each seed stock used or novel genotype generated. Describe any experiments used to assess the effect of a mutation and, where applicable, how potential secondary effects (e.g. second site T-DNA insertions, mosiacism, off-target gene editing) were examined.*
