# Supplementary material for: Novel polymyxin resistance gene family mcr-12 from environmental Pigmentiphaga litoralis
Source: Nat Commun. 2026 Jul 15;17:6187. doi: 10.1038/s41467-026-75587-4 (PMC13373230; doi:10.1038/s41467-026-75587-4)
Supplement: Supplementary file 3 — Transparent Peer Review file [file 41467_2026_75587_MOESM3_ESM.pdf]

nature portfolio

# Peer Review File

# Novel polymyxin resistance gene family mcr-12 from environmental Pigmentiphaga litoralis

Corresponding Author: Professor Amy Cain

Parts of this Peer Review File have been redacted as indicated to remove third-party material.

**This file contains all reviewer reports in order by version, followed by all author rebuttals in order by version.**

Version 0:

Reviewer comments:

Reviewer #1

(Remarks to the Author)
This manuscript describes a novel polymyxin resistance gene, mcr-12.1, identified on a non-conjugative plasmid in Pigmentiphaga litoralis. The authors present a comprehensive characterization of this gene, demonstrating its ability to confer a mild increase in polymyxin resistance in Enterobacterales and a more pronounced effect in P. litoralis and Pseudomonas protegens, both of which already display reduced susceptibility.

The manuscript is well-written, and the discovery of a novel mcr gene outside of the typical clinical or agricultural contexts constitutes a notable advancement. However, several key limitations undermine the manuscript's current suitability for publication in Nature Communications. Specifically, the work lacks mechanistic depth and contains critical errors that must be addressed.
The story of mcr-12.1 parallels that of mcr-9, in that it encodes a phosphoethanolamine transferase with only modest resistance activity, particularly in Enterobacterales. The authors should examine whether mcr-12.1 is inducible, as demonstrated for mcr-9. Additionally, exploring lipid A modification differences between P. litoralis and E. coli could help explain the variable phenotypic impact. Including a mcr-1-positive control strain would provide a valuable reference point. These investigations could form the foundation for a more compelling narrative around host-specific expression and resistance dynamics, which are central to understanding the gene's evolutionary success and public health relevance.

While the gene is novel, it does not confer clinically meaningful resistance in the primary pathogens of concern — Enterobacterales. Assertions such as "significant health implications" (L207) are overstated, given the limited phenotypic impact in clinically relevant species. This should be tempered to reflect the data more accurately.

The statement that mcr-like genes originated from Paenibacillus sophorae is incorrect. As demonstrated in Kieffer et al. (AAC, 2016; PMID: 28320720), the origin of mcr-1/2 lies in Moraxella species. There is no validated evidence supporting the idea that Gram-positive species serve as resistance reservoirs for Gram-negative bacteria. This correction is essential and reflects a broader need for the authors to update their literature references.

Extended Data Table 4 reports an MIC of 200 µg/mL for ampicillin in E. coli carrying the empty pBBR1MCS-2 vector. This is highly implausible unless the vector itself encodes a β-lactamase. The authors must clarify this or use a more appropriate recipient strain. Additionally, the reported MIC for penicillin G (40 µg/mL) is inconsistent with the intrinsic resistance of Gram-negative bacteria to this antibiotic and should be revised or removed.

Minor comments:
-Extended Data Figure 3 would benefit from inclusion of a mcr-1-expressing strain as a positive control to benchmark resistance levels.
-Did the author try codon optimization to enhance the gene effect?
-The claim that Pigmentiphaga is a resistance reservoir (L243–244) is speculative and unsupported.
- The IS1182 element mentioned on lines 262–264 is too distant from mcr-12.1 to suggest any role in mobilization.
-Finally, the stated 59% amino acid identity between OXA-926 and OXA-1383 is too low to support a close phylogenetic relationship (L295).

Reviewer #2

(Remarks to the Author)
This manuscript reports on the finding of a new mcr resistance gene family (mcr-12) from a non-Gammaproteobacterial host. The study is interesting, particularly as it describes the first mcr family discovered outside of a clinical, food or livestock context, outside of Gammaproteobacteria, and in a context where it is located together with metal resistance genes, suggesting the ability for co-selection. The study is well thought through, and the authors interpretations of the results are very well balanced. Methods are accurate, appropriate and well described. The results are solid and all point in the same direction.
I only have two minor comments on the study (see below), which I think deserve being published with two small additions. I recognize that Nature Communications is typically not publishing these novel mcr gene reports, and I will leave the suitability for publishing in this broad-readership journal to the editor. However, I would like to point in that the study is unusually solid and goes to great lengths to investigate the effect that this mcr gene (as well as a predicted beta-lactamase gene also discovered) has on the polymyxin sensitivity in different pathogens. The authors also investigate changes in lipid composition aiding in identifying the mechanism of action of the gene.

Specific comments:
- Lines 291-293: The idea that this would be a very narrow spectrum beta-lactamase seems a bit speculative to me; in my mind it could just be a hitchhiking gene that happens to exist on the same plasmid, despite not conferring a fitness advantage in this specific host. This should be acknowledged as an alternative explanation.
- Line 310: How was the sequence accuracy determined, given the high error rate of NanoPore sequencing? How did the authors make sure that the investigated sequences are actually the correct ones? Please supply more details on this.

Reviewer #3

(Remarks to the Author)
L 30: Is it necessary to have this five lanes here, as it is already well known?
L 40: Same comment as above.
L 44: Please remember genes are genes, and " L-Ara4N transferases such as spmnC ..."
L 57: Why? Do we need to know the origin for proper antimicrobial stewardship?
L 66: How can we predict it will be effectively recruited and disseminated to clinically relevant pathogens? These genes and effector proteins have their own role in P. litoralis,(in this case) for any physiological ecological or any other reason in bacterial economy.
L 71: The authors are not consistent with numbering. As this is , so far, the first an only allele...it should be mcr-12.
L 71: There is not a single reference in the text to the involved bacteria/ Was it isolated by the authors, obtainded from a collection, deposited in a collection, nor referenced to the environment from where it was isolated. Contamination with heavy metals was natural on derived from human activities. Is this environment polluted with human derived bacteria?
L 114: There is a strong need to choose a good denomination for this isolate (or strain if it fulfills the requirements). It is not the species...it is this specific isolate with this plasmid. Are there any other data for any other isolate giving similar results?
L 115: ...(minimal inhibitory concentration of 80 µg/ml) do not use being. The authors assume the break points from E. coli but this is not a clinical relevant bacteria, and the method used for determining the MIC is completely different. Moreover, assuming this could be considered as a correct assumption, the susceptible definition here is too close to the resistance definition for E. coli.
L 137: Is it necessary to try different methods (MIC and diffusion) when MIC would be enough?
Break points for diffusion?
L 162: Just doubling an MIC is difficult to be assumed as significantly different.
L 241: The comment on the potential transfer to gram positive microorganisms is not pertinent, as gram positive microorganisms do not have these genes, just because they do not have outer membrane and, obviously, LPS.
Table 1:
As stated above, it is difficult to sustain any significant difference just by doubling a MIC value.
The authors have used at least two different scale values, one having 1 ug/ml and the other havin 1.248 or 1.25
From this table, only data corresponding to polymixin B, Arsenate (arsenite) and Zinc (4x fold) deserve to be retained. Other data, just in the text.
Figure 1
As already stated, why MCR-12-1? let's start an internal numbering only if other allelic variants appear. In the text there are discrepancies on this.
Figure 3
The relevant point here is the MIC, what other information can be collected form the figures?
t is, at the end, an MIC determined as a standard MIC and these figures? Be consistent.
It looks that polymixin B increases bacterial growth at least for the wild type?
Polymixin B/colistin ...we guess it is the sodium salt, but different considerations should be done by using sulphates.
Figure 4
As already stated before, one dilution indetermination is considered no significant difference.

From these tables, in order to provide an MIC, only P. protegens is providing convincing information.

extended Data Table 3.
What does a 0 diameter mean?

Extended Data Table 4
Please, select a single method for evaluating the difference (MIC or diffusion). In any case, there are no difference, so these data could be better described as text, if retained.
The reported MICs of Penicillin G and even Oxacillin are, somehow, surprising.

All the discussion on data regarding β-lactams susceptibility/resistance is not consistent.

Reviewer #4

(Remarks to the Author)
I co-reviewed this manuscript with one of the reviewers who provided the listed reports. This is part of the Nature Communications initiative to facilitate training in peer review and to provide appropriate recognition for Early Career Researchers who co-review manuscripts.

Version 1:

Reviewer comments:

Reviewer #1

(Remarks to the Author)
The authors have broadly addressed my comments, and the manuscript has improved substantially. In its current form, it provides a thorough characterization of this new mcr determinant, Mcr-12, avoids alarmist interpretations, and maintains an objective tone. Overall, it is a solid and high-quality piece of work.
However, one critical point remains unresolved. I apologize for insisting, but the statement claiming that mcr-1/2 originates from Paenibacillus sophorae is incorrect. I encourage the authors to verify this themselves, as I have: there is no sequence data from P. sophorae containing an mcr gene. This appears to result from an error in the NCBI database. A similar issue occurred previously (observed by myself ten years ago), where an mcr-1-like gene was attributed to Clostridium durum, which was later shown to be a mistake originating from the original mcr-1 publication (it was in fact a misannotated Moraxella). I have personally dedicated time to investigating and correcting these inaccuracies, which is why I insist on this point. I strongly recommend removing this statement to prevent the further dissemination of incorrect information. The fact that this claim is supported by citations is precisely why I consider it particularly important to address.
Therefore, I ask the authors to either remove these citations (to limit the visibility of flawed work) or explicitly clarify the error —although the latter may fall outside the scope of this manuscript.

Reviewer #3

(Remarks to the Author)
[Please see the attached PDF file with formatted text]

Comments from reviewer 3 to Author responses (in italics)

Reviewer 3
- L 30: Is it necessary to have this five lanes here, as it is already well known?
We believe succinct background information on the mechanism of polymyxin antibacterial activity is pertinent and because of the broad readership of Nature Communications, we cannot assume the reader has detailed knowledge in this area. An understanding of this mechanism for the reader is required to understand why the lipid A structure was examined section "MCR-12 is a phosphoethanolamine transferase" and Fig 6. The level of detail here is targeted and appropriate and remains unchanged.

Shorten if possible
-
L 40: Same comment as above.

Same coment as above

L 57: Why? Do we need to know the origin for proper antimicrobial stewardship?

We agree with this point about mcr gene origins and this line has been made clearer "To improve the surveillance of polymyxin resistance genes in environmental reservoirs and inform a One Health approach to antimicrobial stewardship, a broad diversity of mcr genes must be investigated and their resistance mechanisms understood" (L81-84).

However, testing for more genes is against the feasibility for most laboratories. As comparing with another point raised by the authors, would you be testing for , lets say, one hundred OXA genes or testing for relevant OXA genes, incorporating those that start to be relevant when (usually by full genome sequence) still not relevant markers start to be considered an emergent risk?

L 71: There is not a single reference in the text to the involved bacteria/ Was it isolated by the authors, obtained from a collection, deposited in a collection, nor referenced to the environment from where it was isolated. Contamination with heavy metals was natural on derived from human activities. Is this environment polluted with human derived bacteria?
The reviewer is correct, the detail of P. litoralis isolation was not focused on here, as "P. litoralis E30 was isolated from samples obtained from heavy metal contaminated sediment at Crooked Creek in Lake Macquarie (NSW, Australia) in May 2021, as part of a larger study examining antibiotic and heavy metal co-selection14" (L385-387). This other manuscript (reference 14) contains more detail on the strain isolation and has now been published as a BioRxiv pre-print.

We are sorry, but we do not agree in considering a BioRxiv pre-print as a formal publication. However, it might depend on the editorial committee consideration. It might happen that it never reaches formal publication in a peer reviewed journal. Having said that, we still consider a brief description including some isolation and identification details is needed within this manuscript
-

L 241: The comment on the potential transfer to gram positive microorganisms is not pertinent, as gram positive microorganisms do not have these genes, just because they do not have outer membrane and, obviously, LPS.

Reviewer 1 raised the same point; this has been addressed above. In brief, we agree that the function of these genes must differ, however broad range of related enzymes do exist across the bacterial domain (L303-305).

This is a bad interpretation of previous data, just because the authors refer to articles that made erroneous assumptions, and have not verified the sequences validity. We can understand that the first articles describing emergence of plasmid mediated resistance to colistin-could have wrong statements, in the hurry for publication.
However, since one of the the first reviews published by Olaitan and other autors, it must be assumed authors had time for verifying the basis of the cited information, and a letter to the editor was published pointing out that the gene was perhaps assumed to be from paeinibacillus, but the reference sequence even at that time was already discarded from gene bank because it was erroneasly thought to be coming from that origin. 10.1016/j.ijantimicag.2017.06.003
We have re checked this week what is stated in gene bank, and asked NCBI why it was removed with this answer

[REDACTED]

There is not outer membrane, there is not anything related to LPS to be modified, by a protein that has two domains, one immersed in the cytoplasmic membrane and the other facing the typical perisplasmic of gram negative bacteria.

New Table 2
- From this table, only data corresponding to polymixin B, Arsenate (arsenite) and Zinc (4x fold) deserve to be retained. Other data, just in the text.
We understand the rationale for this suggestion, but believe that breaking up the data set between table and text does not serve the reader. Having no change in phenotype is a meaningful and valid result in the context of the study, as it highlights disparities between genotype and phenotype.

We agree that negative results (in this case, no differences) are valid results. Should it had been the case that 90% of the data presented difference and only a few were not different, we could agree. But this is not the case. We still consider presenting the same (no differences) as part of the text will even better highlight those in which there is a difference. We cannot evaluate the statistical meaning of the consensus data as nothing is said.

New Figures 4 / 5- The relevant point here is the MIC, what other information can be collected form the figures? t is, at the end, an MIC determined as a standard MIC and these figures? Be consistent.
We have not removed the figure as the presented kill curve graphs* conveys more information than simply the MIC value. By observing the OD590 over serial dilutions of polymyxin, the reader can appreciate whether the organism is growing to cell density comparable with no antibiotic exposure, or whether growth is somewhat limited by the antibiotic exposure. For example, for P. protegens Pf-5 (Fig 4f) "growth was not impeded until polymyxin B concentrations reached 32 µg/mL" (L190-191), but it is able to withstand* antibiotic exposure still up to 256 µg/mL. A second example is raised by reviewer 3 below

where exposure to sub-MIC polymyxin B displayed increased growth densities at the end of the time period.

- It looks that polymixin B increases bacterial growth at least for the wild type?
This is a curious observation that we are unable to satisfactorily explain at this stage. P. litoralis wild-type exposed to sub-MIC polymyxin B consistently exhibited increased cell density by the end of the time period compared to the unexposed control. A hypothesis for this is discussed in "Curiously, the P. litoralis carrying pPLE30.2 grew to a higher cell density when exposed to sub-MIC concentrations of polymyxin B or colistin (Fig. 4). Exposure to sub-inhibitory antibiotic concentrations can alter the growth kinetics in many bacterial species36, yet the reason(s) underlying this particular fitness response remain unclear and warrant further study" (L307-312).

We have re-reviewed figures 4 and 5, and we really do not understand what the authors mean. These are not killing (or survival) curves, so the explanation that strain X survive up to concentration Z is not valid at all. Survival curves are made by counting CFU/ml during some time. If MICs are evaluated by using these curves, it may be difficult to make interpretations as at some point repetitions give/do not give 0 growth. By rethinking our previous observation, morphological alterations may give differences in OD, that may or not correlate with actual bacterial counts. Having said so, we think it should be better to delete all these results.

Supplementary Data Table2.
- What does a 0 diameter mean?
We acknowledge that a diameter of 0 is unclear, so it has been replaced with "none" (Supplementary Table 2).

Upon closer review, the metric of diameter, recommended by Collins & Lynes Microbiological methods, is inconsistent with the wider literature that measure the annular radius zone of inhibition for the Stokes Test. Therefore, all diameter measurements have been updated to annular radius (Supplementary Tables 2 and 3) and the citation updated to reference 72 (L430).

Both CLSI and EUCAST refer that the absence of any visible diameter around the standard 6 mm disks is reported as 6 mm, just because it is not possible to assure by standard unaided eye examination if there is or not any inhibition zone just by the presence of the disks. Rosco tablets are interpreted differently as the tabets are different in size, as an example. There is no way to compare results that are undefined, therefore if both MICs are defined as >1000, just say that in words.
Regarding the diameters, we cannot assess if the differences between Imipenem results are significant.

Suplemmentary Table 3 (data on E. coli top 10 with or without bla oxa -1383 in the same vector)
Please, select a single method for evaluating the difference (MIC or diffusion). In any case, there are no difference, so these data could be better described as text, if retained.
Although we appreciate that including two methodologies in one table is not the most conventional way of presenting data, in this case, we wanted an efficient way to show that multiple methodologies and multiple antibiotics were trialled when attempting to assign a resistance phenotype for this new blaOXA gene. We believe that splitting the MIC and diffusion into two separate tables would not serve the reader as both tables would convey the same message, and it would be cumbersome to describe in the text, so we firmly recommend that they remain presented together in Supplementary Table 3, under different subpanels.

If the diameter of the disks is 6 mm, how can the authors state a 4/3.3 mm in cephalotin? Again we cannot make any statistical assumption in all data presented here, except for the MICs…all the same.

All the discussion on data regarding β-lactams susceptibility/resistance is not consistent.
We agree that the wording "β-lactam resistance phenotype of OXA-1383 remains unconfirmed over the twelve β-lactams tested here" (Original manuscript lines 291-292), does not convey our intended meaning. The phenotype has been confirmed to be susceptible, the intended meaning was that "the β-lactam substrate of OXA-1383 is not one of the ten β-lactams tested here" (L362-363).

It must be said that several chromosomal OXA enzymes remain to be shown as having relevant betalactamase activity. They are named as OXA just by their homology, they are for other reasons and some of them, in an appropriate (or from human beings stand point) innapropiate platforms and bugs are able to degrade beta lactam antibiotics. Please, consider this point before making any statement.

Reviewer #4

(Remarks to the Author)
I co-reviewed this manuscript with one of the reviewers who provided the listed reports. This is part of the Nature Communications initiative to facilitate training in peer review and to provide appropriate recognition for Early Career Researchers who co-review manuscripts.

**Open Access** This Peer Review File is licensed under a Creative Commons Attribution 4.0 International License, which permits use, sharing, adaptation, distribution and reproduction in any medium or format, as long as you give appropriate credit to the original author(s) and the source, provide a link to the Creative Commons license, and indicate if changes were made.
In cases where reviewers are anonymous, credit should be given to 'Anonymous Referee' and the source.
The images or other third party material in this Peer Review File are included in the article's Creative Commons license, unless indicated otherwise in a credit line to the material. If material is not included in the article's Creative Commons license and your intended use is not permitted by statutory regulation or exceeds the permitted use, you will need to obtain permission directly from the copyright holder.
To view a copy of this license, visit https://creativecommons.org/licenses/by/4.0/

**Author responses to reviewer comments**

We thank the reviewers for their valuable insights on our manuscript. Please see below a point-by-point response to the reviewers' comments and concerns. Line number references by the reviewers refers to the original submitted manuscript, to preserve the quoted comments verbatim. Line numbers in author responses however refer to the revised manuscript file, unless indicated otherwise, with changes in bold for the convenience of the reviewers.

**Reviewer 1**

- *The authors should examine whether mcr-12.1 is inducible*

This was a valuable suggestion from the reviewer, as it is of considerable relevance whether *mcr-12* is constitutively expressed or requires induction in its wild-type host, *Pigmentiphaga litoralis*. We performed additional RT-qPCR expression studies on *P. litoralis* cultured under four sub-MIC levels of polymyxin B, examining levels of *mcr-12* transcript normalised to *rpoB* and *repB*. Methods were consistent with RT-qPCR of *E. coli* endogenous PEtN transferase genes, so relevant methods have been amalgamated (L482-508). The outcome of this experiment confirmed that "***The expression of mcr-12 in pPLE30.2 was not significantly changed after exposure to polymyxin B, over a concentration range of 0.02–2 µg/mL. The constitutive expression of mcr-12 was confirmed by reverse transcription-quantitative PCR (RT-qPCR) with the mcr-12 transcripts normalised to a chromosomal housekeeping gene (rpoB; Supplementary Fig. 3a), or a plasmid copy number control (repB; Supplementary Fig. 3b).***" (Line 142-147). This interpretation has been integrated into the discussion of whether *mcr-12* is co-selected along with metal resistance genes "***Despite their proximity, it is not suggested that they share a regulatory pathway as mcr-12 appears constitutively expressed, consistent with mcr-1, but not mcr-9[49], whereas it is established that czcCBA and arsHCB are regulated by CzcRS[17] and ArsR[50] following ion exposure, respectively***" (L342-345).

- *Additionally, exploring lipid A modification differences between P. litoralis and E. coli could help explain the variable phenotypic impact.*

We thank the reviewer for this informed suggestion. Additional mass spectrometry analysis of lipid A was carried out for *P. litoralis*. "***Comparison of the lipid A spectral profiles between the pPLE30.2-carrying strain and its plasmid-cured counterpart revealed a peak at m/z 1,471 that was unique to the pPLE30.2-carrying strain. This peak is 123 Da greater than the shared m/z 1,348 peak (Fig. 6e-f)***" (L230-233). This confirmed that PEtN addition occurred in the original host.

The "***MCR-12** is a phosphoethanolamine transferase*" results section has been amended to include this data, incorporating the *P. litoralis* spectra after the *E. coli* and *P. protegens* results in a comparison of heterologous and native host PEtN modification (L212-240). We also repeated the *P. protegens* lipid A analysis to allow

a more robust comparison with *P. litoralis*. Methods have added to the existing analyses section, for all three species (L509-526).

Due to the generation of high-quality spectral data from three organisms, original Figure 5 (now as Figure 6) has been restructured to consist solely of the mass spectral data from all three species. The polymyxin B and colistin MIC data for *E. coli* and *mcr-12* RT-qPCR data (originally Fig. 5A-C) has been amalgamated to the rest of the *E. coli* RT-qPCR data as Supplementary Fig. 6.

The mass and therefore structure of the major lipid A peak does differ between the three species, and this is now discussed as a possible explanation for the variable phenotype, "***As expected, core lipid A structure differed among the three analysed species[26], and these structural differences may influence the net ionic effect of PEtN addition.***" (L277-279). Additionally, how the MCR-12 enzyme functions in heterologous hosts is addressed in "***Nonetheless, lipid A analysis confirmed that MCR-12 retained enzymatic functionality even in E. coli. It remains plausible that enzyme kinetics in the heterologous host was diminished, resulting in lower overall levels of PEtN-modified lipid A. Relative quantification of PEtN-modified lipid A may indicate whether this is the case***" (L288-291).

- *Including a mcr-1-positive control strain would provide a valuable reference point… Extended Data Figure 3 would benefit from inclusion of a mcr-1-expressing strain as a positive control to benchmark resistance levels.*

We agree that benchmarking the phenotype of *mcr-12* against *mcr-1* is a useful metric to gauge the relative impact of *mcr-12*. Rather than provide this data in Extended Data Figure 3 (now Supplementary Fig. 4), for clarity, we provided this detail in Figure 5, and we received approval from the editor to make this change.

*mcr-1* was synthesized and cloned into all plasmid constructs, transformed, "***The mcr-1 sequence (GenBank: AKF16168.1) was supplied as a gBlock (Integrated DNA Technologies, Singapore) and cloned using the same methodology as mcr-12.***" (L453-455), and the MICs were measured following the *mcr-12* methodology. We confined this to only strains where *mcr-12* displayed an obvious polymyxin B increase, as performing this in strains where *mcr-12* had no effect would not yield informative conclusions of *mcr-12*. These strains that displayed no MIC change have been relegated to Supplementary Figure 5, though their MIC values are still presented in Table 3. We additionally included *mcr-1* expressed in *E. coli* as the sole representative for strains where *mcr-12*-mediated MIC shifts were negligible, "***expression of mcr-1 produced the anticipated increase in MIC to 4 µg/mL[10]***" (L179-180). This *E. coli* data is presented as Figure 5g, which is slightly different from Supplementary Figure 6a (originally Figure 5a), as in the former, Biolog data is included to be consistent with the rest of the figure panels, whereas in

Supplementary Figure 6a, standard $OD_{600}$ plate reader data is displayed in concordance with Figure 5a in the original manuscript.

The MIC shifts between *mcr-1* and *mcr-12* were comparable in *Pseudomonas* strains and *A. baumannii* ATCC 17978 "***The level of resistance conferred by mcr-12 was identical to that of mcr-1, which was utilised as a reference for clinically relevant mcr genes.***" (L176-178), but for *A. baumannii* BAL062 and Ex003 "***In these additional A. baumannii strains, the MIC conferred by MCR-1 was four-fold greater than that mediated by MCR-12***" (L197-198). This is discussed in terms of *mcr-12* impact of MIC phenotype "***Considering that the clinically relevant mcr-1 produced a similar MIC shift in Pseudomonas, mcr-12 may be comparably effective in conferring lipid A modification and polymyxin resistance in select strains***" (L268-270).

- *Assertions such as "significant health implications" (L207) are overstated, given the limited phenotypic impact in clinically relevant species. This should be tempered to reflect the data more accurately.*

We recognise that this could have an overstated interpretation. Thus, this has been amended to "*detection of a new mcr gene from freshwater sediment should spur on further studies examining the mobility and distribution of polymyxin resistance genes* ***that may possess greater clinical relevance***" (L254-256).

We have also removed lines with could be misinterpreted such as "*The plasmid context of mcr genes bears on their clinical relevance"* (original manuscript line 264-265). Similarly, "*mcr-12 functions more optimally in Psuedomonadales than in Enterobacteriaceae*" (original manuscript L218-219) has been replaced with "***MCR-12 reduces polymyxin susceptibility to a greater degree in Pseudomonadales than in Enterobacteriaceae***" (L264-265).

*- Extended Data Table 4 reports an MIC of 200 µg/mL for ampicillin in E. coli carrying the empty pBBR1MCS-2 vector. This is highly implausible unless the vector itself encodes a β-lactamase. The authors must clarify this or use a more appropriate recipient strain. Additionally, the reported MIC for penicillin G (40 µg/mL) is inconsistent with the intrinsic resistance of Gram-negative bacteria to this antibiotic and should be revised or removed.*

The reviewer is correct and we thank them for pointing out this inconsistency. In response, we have removed the penicillin G data from Supplementary Table 2 and 3, as it is an inappropriate screening antibiotic for Gram-negatives. Further, this comment prompted us to validate the ampicillin phenotype, which is the more relevant penicillin-group antibiotic for Gram-negatives. MIC assays were repeated with freshly remade ampicillin stocks on three biological replicates. The revised MIC with this new antibiotic stock is now consistent with the strains used and has been added to replace the old ampicillin data in Supplementary Table 3 (*E. coli* – 6.25

µg/mL), as well as Supplementary Table 2 (*P. litoralis* – >1000 µg/mL), and associated raw data to the source data file. Removal of this data has been summarized in the reporting summary. Text relating to penicillin G have been removed from the manuscript (L162-164, L362, L422).

- *The statement that mcr-like genes originated from Paenibacillus sophorae is incorrect. As demonstrated in Kieffer et al. (AAC, 2016; PMID: 28320720), the origin of mcr-1/2 lies in Moraxella species.*

We agree that *Moraxella* is a much more likely point of origin than *P. sophorae*, considering the same plasmids carrying *mcr* in *E. coli* are found in *Moraxella*. However, the statement concerning *P. sophorae* is not without foundation. The two cited papers provided evidence of an evolutionary relationship between MCR-1 in *E. coli* and EptA in *P. sophorae*, and suggested that horizontal gene transfer had occurred. We agree that EptA cannot function like MCR-1 in *P. sophorae,* due to differences in cell envelopes, however, the point that similar functional enzymes are distributed across broad phylogenetic range is still valid. It has been rephrased to make this point clearer "***PEtN transferases homologous to MCR-1 and MCR-2 are present in Gram-positive taxa such as Paenibacillus sophorae[10,12], indicating that related enzymes are distributed across a broad phylogenetic range***" (L303-305).

- *Did the author try codon optimization to enhance the gene effect?*

Codon optimization was considered, but decided against in order to model the effect of *mcr-12* being acquired by HGT. The relevance of testing the native sequence has been elaborated in "*Codon usage was not harmonised across host organisms **because our aim was to assess the transferability of the P. litoralis mcr-12 native coding sequence and test its resistance phenotype in clinically-relevant bacteria, thereby modelling what may be the impacts of mcr-12 horizontal gene transfer***" (L284-287).

- *The claim that Pigmentiphaga is a resistance reservoir (L243–244) is speculative and unsupported.*

We agree that the original paragraph structure could be restructured to better support our interpretation. Therefore, the scope of discussion has been refocused on Betaproteobacterium as an overlooked reservoir, with several examples of *mcr* genes potentially transferring between this class and Gammaproteobacteria (L296-324). The explicit reference to *Pigmentiphaga* has been removed.

*- The IS1182 element mentioned on lines 262–264 is too distant from mcr-12.1 to suggest any role in mobilization.*

We agree that the evidence for this involvement in *mcr-12* mobilization is unsubstantiated. However, IS*1182* is directly adjacent to metal resistance genes, so this has been adjusted to "*IS1182 insertion sequence downstream, which may have been involved in the original integration **of metal resistance genes***" (L330-332).

*- Finally, the stated 59% amino acid identity between OXA-926 and OXA-1383 is too low to support a close phylogenetic relationship (L295).*

We agree that "*close phylogeny*" may have been misinterpreted. Line 367 has been amended to *"reinforcing a **possible shared evolution** with OXA-1383"*.

**Reviewer 2**

- *Lines 291-293: The idea that this would be a very narrow spectrum beta-lactamase seems a bit speculative to me; in my mind it could just be a hitchhiking gene that happens to exist on the same plasmid, despite not conferring a fitness advantage in this specific host. This should be acknowledged as an alternative explanation.*

This is a theoretical claim, however there is evidence to support it, namely that "***Its** closest **known** homologue, OXA-926, **has a narrow substrate profile***" (L364-365) described in the reference 22. Alternatively, it may be unrelated to resistance, and this has been acknowledged as ***"Alternatively, bla$_{OXA-1383}$ may not encode a functional resistance enzyme, and its presence may be incidental and not impact fitness of the host"*** (L367-369).

The suggestion that *bla*$_{OXA-1383}$ is not a functional beta-lactamase naturally then requires an explanation of the very low beta-lactam susceptibility of *P. litoralis*. This follows with ***"The maintained high β-lactam resistance despite the removal of pPLE30.2 may be due to alternative β-lactamases, efflux pump[56], or modified penicillin-binding proteins[57]"***(L369-371).

- *Line 310: How was the sequence accuracy determined, given the high error rate of NanoPore sequencing? How did the authors make sure that the investigated sequences are actually the correct ones? Please supply more details on this.*

*Pigmentiphaga litoralis* E30 underwent two independent nanopore sequencing runs. The method detail of this assembly is now provided :"***The complete genome of two P. litoralis colonies were sequenced by Oxford nanopore on R10.4.1 flow cells with basecalling by Guppy v6.5.7. Returned FASTQ reads above 1 kb in length were retained using Filtlong v0.2.1[58] and quality was assessed using Nanoplot v1.36.2[59]. Contigs were assembled with Flye v2.9.3 using automatic minimum overlap between read, metagenomic assembly, and one polishing iteratio[60]"*** (L388-393).

Nanopore sequencing does have a higher error rate relative to Illumina, but this is compensated by "***Both assembled sequences contained pPLE30.2, with 57 and 72 x***

***coverage, respectively***" (L394-395). The current flow cell, guppy basecalling and Flye assembly tool with polishing iterations are recognised as having adequate sequencing accuracy for whole genome sequencing according to reference 61. The two sequence outputs were highly corroborative and any discrepancies were resolved: "***This high sequencing depth, and recent improvements in the accuracy of Oxford Nanopore sequencing[61], produced 40 single or dinucleotide polymorphisms, which were reconciled depending on whether they occurred in coding or non-coding regions. For coding regions, the nucleotide that was most consistent with codon usage for P. litoralis was retained. Codon usage was calculated by quantifying codon proportions across all open reading frames. All nucleotide polymorphisms occurring outside coding regions occurred within mononuclear repeats (>4 Ns); in these cases, the longer repeat was retained, as Oxford Nanopore with Guppy base calling tends to delete bases in homopolymeric regions[62]***" (L395-403).

For the *mcr-12* and *bla*$_{\text{OXA-1383}}$ genes, their DNA sequences were further verified during the expression vector construction "*construct sequences were verified by* ***Sanger*** *sequencing*" (L455-456).

**Reviewer 3**

- *L 30: Is it necessary to have this five lanes here, as it is already well known?*

We believe succinct background information on the mechanism of polymyxin antibacterial activity is pertinent and because of the broad readership of Nature Communications, we cannot assume the reader has detailed knowledge in this area. An understanding of this mechanism for the reader is required to understand why the lipid A structure was examined section "***MCR-12*** *is a phosphoethanolamine transferase*" and Fig 6. The level of detail here is targeted and appropriate and remains unchanged.

- *L 40: Same comment as above.*

As per the L30 comment, this background is about spontaneous polymyxin resistance mechanisms. This line links how resistance mechanisms influence the lipid A structure. This information is needed to be understood by the reader to appreciate why we investigated alternative endogenous resistance genes in "***MCR-12*** *is a phosphoethanolamine transferase*" and Supplementary Fig 6.

- *L 44: Please remember genes are genes, and " L-Ara4N transferases such as pmrC ..."*

We thank the reviewer for spotting this oversight, we have corrected it to "*L-Ara4N transferase* ***genes*** *such as pmrC"* (L67-68).
Similar mistakes in discussing genes and protein comparisons have been rectified at:
*"revealed a novel potential resistance gene located between czcCBA and cusRS (Fig. 1b;* ***bases 130397–132043****) with* ***its product displaying*** *39.3% amino acid identity* ***compared*** *to the polymyxin resistance determinant EptA"* (L111-114)
*"distinct gene lineage from that of the chromosomal* ***eptA"*** (L120-121)
"novel **protein** was more closely related to MCR-5.1" (L123-124)
*"The structural similarity of the mcr-5 and eptA gene* ***products"*** (L137)
*"It encoded a protein with 59% amino acid identity to* ***OXA-926"*** (L158-159)

- *L 57: Why? Do we need to know the origin for proper antimicrobial stewardship?*

We agree with this point about *mcr* gene origins and this line has been made clearer "***To improve the surveillance of polymyxin resistance genes in environmental reservoirs and inform a One Health approach to antimicrobial stewardship, a broad*** *diversity of mcr genes must be investigated and their resistance mechanisms understood*" (L81-84).

- L 66: How can we predict it will be effectively recruited and disseminated to clinically relevant pathogens? These genes and effector proteins have their own role in P. litoralis,(in this case) for any physiological ecological or any other reason in bacterial economy.

We agree that the phrasing in this line could be improved. Rather than be interpreted as a prediction, this has been rephrased as a statement: "*This study provides rare insights into a new potential genetic reservoir for last-resort antibiotic resistance in the environment* ***that has not yet*** *spread to clinically relevant pathogens*" (L91-93).

- *L 71 and Figure 1: The authors are not consistent with numbering. As this is, so far, the first an only allele...it should be mcr-12.*

Naming of novel polymyxins resistance genes is administered by NCBI, and they had assigned the name *mcr-12.1* to this specific gene. We had used *mcr-12.1* to refer to this specific gene, and *mcr-12* when discussing the gene family in comparison to other *mcr* families. However, we agree that ".1" is superfluous at present as there is only one gene, and so we have simplified the gene name to *mcr-12* throughout.

Further, some select references to "family" have been removed where it could be misinterpreted that several *mcr-12* variants have been identified: *"This study characterised a new polymyxin resistance gene* ~~***family***~~*, mcr-12"* (L242) and "*the detection*

*of a new ~~family of~~ mcr gene~~s~~ from freshwater sediment*" (L254). Similarly, the number of *mcr-12* genes has been made clearer in "*our study is the first to experimentally characterise a multiple heavy metal resistance region with* ***an*** *mcr gene~~s~~*" (L346-348)

- *L 71: There is not a single reference in the text to the involved bacteria/ Was it isolated by the authors, obtained from a collection, deposited in a collection, nor referenced to the environment from where it was isolated. Contamination with heavy metals was natural on derived from human activities. Is this environment polluted with human derived bacteria?*

The reviewer is correct, the detail of *P. litoralis* isolation was not focused on here, as ***"P. litoralis E30 was isolated from samples obtained from heavy metal contaminated sediment at Crooked Creek in Lake Macquarie (NSW, Australia) in May 2021, as part of a larger study examining antibiotic and heavy metal co-selection[14]"*** (L385-387). This other manuscript (reference 14) contains more detail on the strain isolation and has now been published as a BioRxiv pre-print.

- *L 114: There is a strong need to choose a good denomination for this isolate (or strain if it fulfills the requirements). It is not the species...it is this specific isolate with this plasmid. Are there any other data for any other isolate giving similar results?*

We agree with the comment. The isolate in question has an isolate designation of "***E30***", and this isolate number has been added into this line (L96), and other direct references to the isolate throughout the manuscript.

- L 115: *...(minimal inhibitory concentration of 80 µg/ml) do not use being. The authors assume the break points from E. coli but this is not a clinical relevant bacteria, and the method used for determining the MIC is completely different. Moreover, assuming this could be considered as a correct assumption, the susceptible definition here is too close to the resistance definition for E. coli.*

We agree the phrasing here could be improved, for clarity this has been updated to "*highly resistant to polymyxin B with a minimum inhibitory concentration (MIC)* ***of*** *80 µg/ml*" (L138-139).

We completely agree with the reviewer that using *E. coli* susceptibility breakpoints would be inappropriate in this context, and we have not used these breakpoints in our interpretation. Rather, we use the microbiological definition outlined by EUCAST, stated in lines 428-430: "*A "change in resistance" in this study refers to its microbiological definition as the acquisition of a specific genetic resistance mechanism impacting a susceptibility phenotype[73]"*.

*- L 137: Is it necessary to try different methods (MIC and diffusion) when MIC would*

*be enough?*
*Break points for diffusion?*
We appreciate the "*and*" made this ambiguous. This line has been corrected to "*as tested by MIC **or** Stokes disc diffusion*" (L166).

*- L 162 and Table 1: Just doubling an MIC is difficult to be assumed as significantly different.*
We agree with this statement, and we do not make the case that a doubling of MIC is statistically significant. However, we added text to emphasize that "***Although the MIC difference observed here is modest, the consistent two-fold decrease in susceptibility across all Acinetobacter and Pseudomonas strains tested here demonstrates phenotypic reproducibility***" (L265-268).

- *L 241: The comment on the potential transfer to gram positive microorganisms is not pertinent, as gram positive microorganisms do not have these genes, just because they do not have outer membrane and, obviously, LPS.*

Reviewer 1 raised the same point; this has been addressed above. In brief, we agree that the function of these genes must differ, however broad range of related enzymes do exist across the bacterial domain (L303-305).

Table 1:
*- The authors have used at least two different scale values, one having 1 ug/ml and the other havin 1.248 or 1.25*
We thank the reviewer for pointing out inconsistencies in the number of significant figures. All tables have been updated so all values have a consistent number of significant figures (Table 1-3, Supplementary Table 2-3).

Furthermore, we have independently corrected the use of negative fold changes, which is nonsensical. The fold-change numbers are now positive values, and heading corrected to "***fold-reduction***" (L891). Due to the standardisation of positive values, the "+" signs in Table 3 are superfluous and have been removed.

*- From this table, only data corresponding to polymixin B, Arsenate (arsenite) and Zinc (4x fold) deserve to be retained. Other data, just in the text.*
We understand the rationale for this suggestion, but believe that breaking up the data set between table and text does not serve the reader. Having no change in phenotype is a meaningful and valid result in the context of the study, as it highlights disparities between genotype and phenotype.

Figure 3

*- The relevant point here is the MIC, what other information can be collected form the figures?*

*t is, at the end, an MIC determined as a standard MIC and these figures? Be consistent.*

We have not removed the figure as the presented kill curve graphs conveys more information than simply the MIC value. By observing the $OD_{590}$ over serial dilutions of polymyxin, the reader can appreciate whether the organism is growing to cell density comparable with no antibiotic exposure, or whether growth is somewhat limited by the antibiotic exposure. For example, for *P. protegens* Pf-5 (Fig 4f) *"growth was not impeded until polymyxin B concentrations reached 32 µg/mL*" (L190-191), but it is able to withstand antibiotic exposure still up to 256 µg/mL. A second example is raised by reviewer 3 below where exposure to sub-MIC polymyxin B displayed increased growth densities at the end of the time period.

*- It looks that polymixin B increases bacterial growth at least for the wild type?*

This is a curious observation that we are unable to satisfactorily explain at this stage. *P. litoralis* wild-type exposed to sub-MIC polymyxin B consistently exhibited increased cell density by the end of the time period compared to the unexposed control. A hypothesis for this is discussed in ***"Curiously, the P. litoralis carrying pPLE30.2 grew to a higher cell density when exposed to sub-MIC concentrations of polymyxin B or colistin (Fig. 4). Exposure to sub-inhibitory antibiotic concentrations can alter the growth kinetics in many bacterial species[36], yet the reason(s) underlying this particular fitness response remain unclear and warrant further study***" (L307-312).

- *Polymixin B/colistin ...we guess it is the sodium salt, but different considerations should be done by using sulphates.*

The use of sulfate salt, in accordance with recommended MIC testing for polymyxin, is stated in the methods "*MICs towards polymyxin B or colistin (as sulfate salts)*" (L472). We believe this additional detail is not required to interpret Tables 2, and 3, Figures 4 and 5, and Supplementary Figures 3, 4, 5 and 6 and adding it would confound the x-axis scale, which is based on the polymyxin component only.

Figure 4

*- As already stated before, one dilution indetermination is considered no significant difference. From these tables, in order to provide an MIC, only P. protegens is providing convincing information.*

Please refer to our response to the previous Reviewer 3 comment regarding two-fold changes in MIC. In brief, two-fold changes are reproducible and are an acceptable metric interval for polymyxin susceptibility. (L265-268)

extended Data Table 3.

*- What does a 0 diameter mean?*

We acknowledge that a diameter of 0 is unclear, so it has been replaced with "***none***" (Supplementary Table 2).

Upon closer review, the metric of diameter, recommended by Collins & Lynes Microbiological methods, is inconsistent with the wider literature that measure the annular radius zone of inhibition for the Stokes Test. Therefore, all diameter measurements have been updated to annular radius (Supplementary Tables 2 and 3) and the citation updated to **reference 72** (L430).

Extended Data Table 4

*Please, select a single method for evaluating the difference (MIC or diffusion). In any case, there are no difference, so these data could be better described as text, if retained.*

Although we appreciate that including two methodologies in one table is not the most conventional way of presenting data, in this case, we wanted an efficient way to show that multiple methodologies and multiple antibiotics were trialled when attempting to assign a resistance phenotype for this new $\textit{bla}_{OXA}$ gene. We believe that splitting the MIC and diffusion into two separate tables would not serve the reader as both tables would convey the same message, and it would be cumbersome to describe in the text, so we firmly recommend that they remain presented together in Supplementary Table 3, under different subpanels.

*The reported MICs of Penicillin G and even Oxacillin are, somehow, surprising.*

As part of our response to related comment on beta-lactams made by reviewer 1, the penicillin G data has been removed (Supplementary Table 2 and 3). In response to this comment, we agree that oxacillin was an inappropriate choice for screening, as it is only clinically used and active against Gram-positive organisms (Wouters et al., 2023, 10.1016/j.bbamem.2023.184172). Therefore, the oxacillin data has been removed from Supplementary Table 2 and 3, and references to oxacillin and penicillin removed throughout the manuscript (L162-164, L362, L422).

*All the discussion on data regarding β-lactams susceptibility/resistance is not consistent.*

We agree that the wording "*β-lactam resistance phenotype of OXA-1383 remains unconfirmed over the twelve β-lactams tested here*" (Original manuscript lines 291-292), does not convey our intended meaning. The phenotype has been confirmed to be susceptible, the intended meaning was that "the *β-lactam substrate of OXA-1383* ***is not one of*** *the* ***ten*** *β-lactams tested here*" (L362-363).

**Additional changes:**

In addition to the changes made to address the author concerns, we would like to make transparent some minor changes we have made to improve accuracy to the manuscript:

1. Since initial submission, *Acinetobacter baumannii* Ex003 pVLT33 was generated, which allowed a direct comparison between *A. baumannii* Ex003 pVLT33-*mcr-12* and its vector control, rather than comparisons against the wild type strain. All relevant data (Fig 5c, Table 3, Supplementary Fig 4c, and Supplementary Fig 7c) has been updated with this vector control, though there is no perceivable difference compared to the results previously generated with the wild-type.
2. Table 2 and Supplementary Table 2: The original Table 1 (now Table 2) displayed MICs of *Pigmentiphaga litoralis* for polymyxin, beta-lactams and heavy metals, whereas the original Extended Table 3 (now Supplementary Table 2) displayed stokes disc diffusion results from *P. litoralis* for beta-lactams. Upon review, it is far simpler for the reader to amalgamate all beta-lactam results from Table 2 into Supplementary Table 2. The beta-lactam results are discussed in a separate section of the manuscript to polymyxin and heavy metals, and in addition, there is a difference in MIC between *P. litoralis* strains for polymyxin and heavy metals, but no change for any beta-lactam irrespective of whether it was measured by MIC or Stokes test.
3. Language referencing RT-qPCR data has been changed from examining "*expression*" to the more accurate "***transcription***" (L206-207), as this data technique yields no direct data on translation.
4. With the additional mass-spectrometry results for *Pigmentiphaga litoralis*, we have condensed the existing paragraph concerning *E. coli* and *P. protegens*, without the loss of the any crucial information (L212-227).
5. The second discussion paragraph (L257-295) has been broken into two and some wording changed to adjust for the paragraph flow, to make it easier for the reader to digest.
6. The plasmid conjugation method (L431-439) and plasmid curing (L416-430) method have been reordered, as the conjugation requires the curing to be performed first to generate the recipient strain.
7. References to "*wild-type*" in the context of *P. litoralis*, have been replaced with "***plasmid-carrying***", (L419, L511, L876, L887, L891) as it may not be intuitive to the reader that the "wild-type" actually contains the plasmid.
8. Plasmids and chromosomes do not "*encode"* anything, they carry genes. This has been rectified to *"plasmid-**borne**"* or similar at L67, L117, L119-121.
9. NCBI has updated its record of OXA-1383 to "***WP_407792312***" (L161), from the previous interim record of *"XFF02152.1"* (putative OXA-926).
10. The convention for mass spectrometry data is for the *m/z* unit to precede the value. This has been updated throughout L221-237.

11. Table 1- Repeated sequence analysis revealed there are minor inaccuracies in the reported query coverage for some of the sequences. This may be due to updated reference sequences or transcription error, in any case these inaccuracies have been rectified.
12. Two primers (BFG25 and BFG26) have been removed from Supplementary Table 4. These do not relate directly to any of the experiments in this manuscript.
13. In Figure 4 and 5, the colour of the green coloured icons has changed, to accommodate for red-green colour blindness, although note that each icon in each figure was also different. The colour and shape of the original green triangles in Figures 4 and 5 are now each different colours and icons so as to not suggest a relationship between the two data sets.
14. Several minor instances where grammar has been adjusted for clarity, these are highlighted in colour in the provided document.

**Author response to reviewers comments, second revision**
We extend our thanks once again to the reviewers for their time and valuable recommendations. Please find attached our point-by-point responses to the reviewers' comments. Line numbers in the author responses refer to the manuscript file of the second (most up to date) revision, unless indicated otherwise, with changes in bold for the convenience of the reviewers.

Reviewer #1 (Remarks to the Author):
The authors have broadly addressed my comments, and the manuscript has improved substantially. In its current form, it provides a thorough characterization of this new mcr determinant, Mcr-12, avoids alarmist interpretations, and maintains an objective tone. Overall, it is a solid and high-quality piece of work.

However, one critical point remains unresolved. I apologize for insisting, but the statement claiming that mcr-1/2 originates from Paenibacillus sophorae is incorrect. I encourage the authors to verify this themselves, as I have: there is no sequence data from P. sophorae containing an mcr gene. This appears to result from an error in the NCBI database. A similar issue occurred previously (observed by myself ten years ago), where an mcr-1-like gene was attributed to Clostridium durum, which was later shown to be a mistake originating from the original mcr-1 publication (it was in fact a misannotated Moraxella). I have personally dedicated time to investigating and correcting these inaccuracies, which is why I insist on this point. I strongly recommend removing this statement to prevent the further dissemination of incorrect information. The fact that this claim is supported by citations is precisely why I consider it particularly important to address.
Therefore, I ask the authors to either remove these citations (to limit the visibility of flawed work) or explicitly clarify the error—although the latter may fall outside the scope of this manuscript.

We thank the reviewer for this valuable information and agree with the reviewer. Accordingly, we have completely removed this statement (first revision manuscript L303-305). Its retention in the first round of revision was due to our misinterpretation of the original criticism, as we understood it to be a request for clarification regarding either a specific *mcr* gene in *P. sophorae* or a broader phosphoethanolamine (PEtN) transferase with functions beyond lipid A modification. We now recognise that this interpretation was incorrect and apologize for the oversight.

Comments from reviewer 3 to Author responses (in italics)

Reviewer 3

- L 30: Is it necessary to have this five lanes here, as it is already well known?

*We believe succinct background information on the mechanism of polymyxin antibacterial activity is pertinent and because of the broad readership of Nature Communications, we cannot assume the reader has detailed knowledge in this area. An understanding of this mechanism for the reader is required to understand why the lipid A structure was examined section "MCR-12 is a phosphoethanolamine transferase" and Fig 6. The level of detail here is targeted and appropriate and remains unchanged.*

We agree that a detailed description of polymyxin activity is not required at this point in the manuscript. However, we consider a brief explanation necessary because it provides the context for our subsequent analysis of lipid A structure (section "MCR-12 is a phosphoethanolamine transferase" and current Fig. 5). To address the reviewer's concern, we have now reduced this background section from 44 to 29 words while retaining only the information essential for understanding the rationale of the study. The revised text now reads: "***Polymyxin antibiotics are bactericidal** against Gram-negative bacteria **by displacing divalent cations ionically bound** to the lipid A phosphate component of the lipopolysaccharide or lipooligosaccharide, **thereby destabilising outer membrane integrity***" (L54–57).

-

L 40: Same comment as above.

Similarly to the above comment, we have reduced this from 43 words to 25 words: "*Polymyxin resistance was traditionally considered to be mediated by **non-transferable and potentially unstable**[8] mutations in transcriptional regulators of chromosomal PEtN transferases and L-Ara4N transferase genes*" (L64-67).

L 57: Why? Do we need to know the origin for proper antimicrobial stewardship?

*We agree with this point about mcr gene origins and this line has been made clearer "To improve the surveillance of polymyxin resistance genes in environmental reservoirs and inform a One Health approach to antimicrobial stewardship, a broad diversity of mcr genes must be investigated and their resistance mechanisms understood" (L81-84).*

However, testing for more genes is against the feasibility for most laboratories. As comparing with another point raised by the authors, would you be testing for, let's say, one hundred OXA genes or testing for relevant OXA genes, incorporating those that start to be relevant when (usually by full genome sequence) still not relevant markers start to be considered an emergent risk?

We agree that expanding routine testing for additional resistance genes is not a practical solution, and this is not the message we are attempting to convey. Rather, we wish to emphasize the importance of initial characterisation of *mcr* diversity in environmental reservoirs to better appreciate the extent of gene dissemination. This

has been clarified as "***To appreciate the extent of mobile polymyxin resistance determinants within a One Health framework, it is important to characterise the diversity of mcr genes present in environmental reservoirs. This provides critical context for gene dissemination and may inform which resistance determinants should be prioritised for future surveillance***" (L79-83).

L 71: There is not a single reference in the text to the involved bacteria/ Was it isolated by the authors, obtained from a collection, deposited in a collection, nor referenced to the environment from where it was isolated. Contamination with heavy metals was natural on derived from human activities. Is this environment polluted with human derived bacteria?

*The reviewer is correct, the detail of P. litoralis isolation was not focused on here, as "P. litoralis E30 was isolated from samples obtained from heavy metal contaminated sediment at Crooked Creek in Lake Macquarie (NSW, Australia) in May 2021, as part of a larger study examining antibiotic and heavy metal co-selection14" (L385-387). This other manuscript (reference 14) contains more detail on the strain isolation and has now been published as a BioRxiv pre-print.*

We are sorry, but we do not agree in considering a BioRxiv pre-print as a formal publication. However, it might depend on the editorial committee consideration. It might happen that it never reaches formal publication in a peer reviewed journal. Having said that, we still consider a brief description including some isolation and identification details is needed within this manuscript.

More details have been included providing more context on the isolation, including the sediment characteristics and coordinates, and identification strategy. There is no longer a reliance on the preprint reference, so both citations at L98 and L387 have been removed.

An additional small paragraph now leads the results section to report on the isolation and identification of *P. litoralis* "***A yellow pigmented isolate (E30), tolerant to at least 0.5 mM cadmium acetate in R2A agar, was cultured from heavy metal contaminated freshwater sediment*** *as part of a broader assessment of Australian heavy metal and antibiotic resistant environmental isolates.* ***Whole genome sequencing and alignment of the entire 16S gene revealed 99.66% identity to P. litoralis JSM 061001 (GenBank: NR_044530.1)***"(L95-99). The section header has been amended to reflect this "*mcr-12* is carried by a multiple metal resistance plasmid **in *Pigmentiphaga litoralis*"** (L94).

The methods now additionally provide extra detail on this location and identification process, warranting a change to the subheading: "***Isolation of Pigmentiphaga litoralis and** characterisation of pPLE30.2*" (L382). The paragraph is now as follows: "*P. litoralis E30 was **isolated from freshwater sediment and cultured at ambient temperature on R2A agar supplemented with 0.5 mM cadmium acetate. Sediment was obtained from the top 50 mm layer of** heavy metal contaminated sediment at Crooked Creek, **adjacent to the Eraring Power Station coal ash settling basin** in Lake Macquarie, NSW, Australia **(33°3'41" S, 151°32'52" E)** in May 2021. **P. litoralis DNA was extracted using a cetyltrimethylammonium bromide method[57]***" (L383-388). In terms of identification subsequent to whole genome sequencing: "***The 16S rRNA gene was identified by barrnap[61] and aligned with the NCBI core nucleotide database, limiting to sequences from type-strain***" (L394-395).

L 241: The comment on the potential transfer to gram positive microorganisms is not pertinent, as gram positive microorganisms do not have these genes, just because they do not have outer membrane and, obviously, LPS.

*Reviewer 1 raised the same point; this has been addressed above. In brief, we agree that the function of these genes must differ, however broad range of related enzymes do exist across the bacterial domain (L303-305).*

This is a bad interpretation of previous data, just because the authors refer to articles that made erroneous assumptions, and have not verified the sequences validity. We can understand that the first articles describing emergence of plasmid mediated resistance to colistin-could have wrong statements, in the hurry for publication.

However, since one of the first reviews published by Olaitan and other authors, it must be assumed authors had time for verifying the basis of the cited information, and a letter to the editor was published pointing out that the gene was perhaps assumed to be from Paeinibacillus, but the reference sequence even at that time was already discarded from gene bank because it was erroneously thought to be coming from that origin. 10.1016/j.ijantimicag.2017.06.003

We have re checked this week what is stated in Genbank, and asked NCBI why it was removed with this answer

**[REDACTED]**

There is not outer membrane, there is not anything related to LPS to be modified, by a protein that has two domains, one immersed in the cytoplasmic membrane and the other facing the typical perisplasmic of gram negative bacteria.

We entirely agree with the reviewer and appreciate the length that they have undertaken to verify this. As explained to a similar comment by reviewer one, the reason this was not properly rectified from the initial submission was because the initial criticism was thought to be one of clarity, that the relevant gene in *P. sophorae* is a non-*mcr* PEtN transferase gene, rather than outright error. We have removed this statement (first revision manuscript L303-305) and thank the reviewer for alerting us to this error.

New Table 2

- From this table, only data corresponding to polymixin B, Arsenate (arsenite) and Zinc (4x fold) deserve to be retained. Other data, just in the text.

*We understand the rationale for this suggestion, but believe that breaking up the data set between table and text does not serve the reader. Having no change in phenotype is a meaningful and valid result in the context of the study, as it highlights disparities between genotype and phenotype.*

We agree that negative results (in this case, no differences) are valid results. Should it had been the case that 90% of the data presented difference and only a few were not different, we could agree. But this is not the case. We still consider presenting the same (no differences) as part of the text will even better highlight those in which there is a difference. We cannot evaluate the statistical meaning of the consensus data as nothing is said.

We have complied with the reviewer suggestion and have removed the cadmium, copper, and cobalt data from Table 2. The results for the metals have been added as a new sentence "***Plasmid curing resulted in a smaller two-fold decrease in tolerance to both cobalt and cadmium (MIC reduced from 2 mM for the plasmid-carrying strain to 1 mM for the plasmid-cured strain), and copper tolerance remained unchanged (MIC of 5 mM)***" (L151-154).

Importantly, we have retained the raw data for copper, cadmium and cobalt in the source data file as belonging to Table 2.

In addition, to make clearer the reproducibility of these results and hence the interpretability, we have added the number of technical replicates in addition to biological replicates for all MIC test results in the legends for Tables 2 and 3, Figure 4, Supplementary Figures 3-6, and Supplementary Table 2 and 3.

New Figures 4 / 5- The relevant point here is the MIC, what other information can be collected form the figures? t is, at the end, an MIC determined as a standard MIC and these figures? Be consistent.

*We have not removed the figure as the presented kill curve graphs* conveys more information than simply the MIC value. By observing the OD590 over serial dilutions of polymyxin, the reader can appreciate whether the organism is growing to cell density comparable with no antibiotic exposure, or whether growth is somewhat limited by the antibiotic exposure. For example, for P. protegens Pf-5 (Fig 4f) "growth was not impeded until polymyxin B concentrations reached 32 µg/mL" (L190-191), but it is able to withstand* antibiotic exposure still up to 256 µg/mL. A second example is raised by reviewer 3 below where exposure to sub-MIC polymyxin B displayed increased growth densities at the end of the time period.*

- It looks that polymixin B increases bacterial growth at least for the wild type?

*This is a curious observation that we are unable to satisfactorily explain at this stage. P. litoralis wild-type exposed to sub-MIC polymyxin B consistently exhibited increased cell density by the end of the time period compared to the unexposed control. A hypothesis for this is discussed in "Curiously, the P. litoralis carrying pPLE30.2 grew to a higher cell density when exposed to sub-MIC concentrations of polymyxin B or colistin (Fig. 4). Exposure to sub-inhibitory antibiotic concentrations can alter the growth kinetics in many bacterial species36, yet the reason(s) underlying this particular fitness response remain unclear and warrant further study" (L307-312).*

We have re-reviewed figures 4 and 5, and we really do not understand what the authors mean. These are not killing (or survival) curves, so the explanation that strain X survives up to concentration Z is not valid at all. Survival curves are made by counting CFU/ml during some time. If MICs are evaluated by using these curves, it may be difficult to make interpretations as at some point repetitions give/do not give 0 growth. By rethinking our previous observation, morphological alterations may give differences in OD, that may or not correlate with actual bacterial counts. Having said so, we think it should be better to delete all these results.

We thank the reviewer for this important clarification. We agree that the original Figures 4 and 5 represented optical density ($OD_{590}$) readings rather than killing or survival curves. Importantly, we do not refer to these as kill curves in the manuscript. We have now revised both the original Figure 4 and Figure 5 accordingly to avoid any ambiguity in interpretation.

We also agree that presentation of the MIC data in the original figures format made interpretation unnecessarily difficult. In response to the reviewers' suggestions, we have now replaced Figure 4 with a column graph showing MIC values, with independent biological replicates overlayed as a dot plot. This allows an easier

interpretation and comparison between strains. For Figure 5, the relevant MIC data are already presented in Table 3; therefore Figure 5 has been removed and all corresponding references have been also updated to refer Table 3 instead. The *mcr*-1 control data have also been added to Table 3.

Consistent with these revisions Supplementary Figure 6a and 6b have been replaced with equivalent MIC column graph, and Supplementary Figure 5 has been removed as this data is reported in Table 3.

We have additionally removed statements discussing apparent increased growth at sub-inhibitory polymyxin concentrations (first revision manuscript L189-191 and L307-312).

The Source file has been updated to reflect these changes as well. Absorbance data previously used for Figure 4 and 5 have been relocated to complement MIC data for the updated Figure 4, Table 3, and Supplementary Figure 6a and 6b.

Supplementary Data Table2.

- What does a 0 diameter mean?

*We acknowledge that a diameter of 0 is unclear, so it has been replaced with "none" (Supplementary Table 2).*

*Upon closer review, the metric of diameter, recommended by Collins & Lynes Microbiological methods, is inconsistent with the wider literature that measure the annular radius zone of inhibition for the Stokes Test. Therefore, all diameter measurements have been updated to annular radius (Supplementary Tables 2 and 3) and the citation updated to reference 72 (L430).*

Both CLSI and EUCAST refer that the absence of any visible diameter around the standard 6 mm disks is reported as 6 mm, just because it is not possible to assure by standard unaided eye examination if there is or not any inhibition zone just by the presence of the disks. Rosco tablets are interpreted differently as the tabets are different in size, as an example. There is no way to compare results that are undefined, therefore if both MICs are defined as >1000, just say that in words.

Regarding the diameters, we cannot assess if the differences between Imipenem results are significant.

We thank the reviewer for highlighting the need for greater clarity in the presentation of Supplementary Tables 2 and 3. We agree that reporting a value of "0" was unclear and potentially misleading. Accordingly, these entries were replaced with "none". "***Modified Stokes' method***" has been added to Supplementary Tables 2 and 3 subheadings, and made clear in the methods and results (L170-171, L428, L472).

As noted previously (in the first revision reporting), reporting inhibition zone diameter was not the most appropriate metrics for the modified Stokes' method. Following the broader modified Stokes' method literature, all measurements in Supplementary

Tables 2 and 3 have therefore been revised to report annular radius values rather than inhibition zone diameters, and hence measurements <6 mm are valid.

We additionally recognise the reviewer's concern regarding interpretation of differences between strains. Therefore, a clarification from the methods (first revised manuscript L427–428) has been added to the footnotes of Supplementary Tables 2 and 3 stating that, "***A paired difference in inhibition zones > 3 mm represents a significant difference***[12]**"**. This criterion provides the basis for interpreting differences observed between strains.

Supplementary Table 3 (data on E. coli top 10 with or without bla oxa -1383 in the same vector)

Please, select a single method for evaluating the difference (MIC or diffusion). In any case, there are no difference, so these data could be better described as text, if retained.

*Although we appreciate that including two methodologies in one table is not the most conventional way of presenting data, in this case, we wanted an efficient way to show that multiple methodologies and multiple antibiotics were trialled when attempting to assign a resistance phenotype for this new blaOXA gene. We believe that splitting the MIC and diffusion into two separate tables would not serve the reader as both tables would convey the same message, and it would be cumbersome to describe in the text, so we firmly recommend that they remain presented together in Supplementary Table 3, under different subpanels.*

If the diameter of the disks is 6 mm, how can the authors state a 4/3.3 mm in cephalotin? Again we cannot make any statistical assumption in all data presented here, except for the MICs…all the same.

This comment relates to the use and interpretation of the modified Stokes' method. As noted in the previous response, results are reported as annular radius rather than inhibition zone diameter. Because annular radius is measured from edge of the antibiotic disk, values <6 mm are valid and reflect measurable inhibition relative to the 6 mm disk size. Accordingly, the values of 4 mm and 3.3 mm for cephalothin represent measurable annular radii of inhibition relative to the edge of the 6 mm disk.

We also agree that statistical inference is not appropriate for the diffusion-based data presented, and no statistical comparisons have been applied to these measurements. Interpretation is instead based on established modified Stokes' method criteria. To improve clarity, these interpretation guidelines have been added to the footnotes of Supplementary Tables 2 and 3.

All the discussion on data regarding β-lactams susceptibility/resistance is not consistent.

*We agree that the wording "β-lactam resistance phenotype of OXA-1383 remains unconfirmed over the twelve β-lactams tested here" (Original manuscript lines 291-292), does not convey our intended meaning. The phenotype has been confirmed to be susceptible, the intended meaning was that "the β-lactam substrate of OXA-1383 is not one of the ten β-lactams tested here" (L362-363).*

It must be said that several chromosomal OXA enzymes remain to be shown as having relevant betalactamase activity. They are named as OXA just by their homology, they are for other reasons and some of them, in an appropriate (or from human beings stand point) inappropriate platforms and bugs are able to degrade beta lactam antibiotics. Please, consider this point before making any statement.

We agree with the reviewer that many beta-lactams are classified on homology alone, and this was alluded to as "*Alternatively, $bla_{OXA-1383}$ may not encode a functional resistance enzyme*" (first revision manuscript L367-368). This point has been made more salient "***placing OXA-1383 among a growing group of homology-predicted OXA-domain enzymes for which substrate specificity remains unknown***[54]" (L357-359). The inconsistency of using OXA-926 to predict the substrate profile of OXA-1383, despite the limitations of homology based prediction for class D beta-lactamase, has also been resolved**:** "***Substantial amino acid heterogeneity within the OXA family complicates functional prediction***[54]***; notably, the substrate profile of OXA-1383 differs from its closest known homologue, OXA-926, in respect to piperacillin and cephalothin***[21]" (L359-362).

We also agree with the reviewer that there is a possibility that induction conditions were not appropriate for gene expression and enzyme activity, and so the modality of the conclusion of the substrate test has been reduced "*The β-lactam substrate* ***profile*** *of OXA-1383* ***appears not to include any*** *of the ten β-lactams tested here*" (L356-357). Possible explanations for keeping these beta-lactams in consideration as substrates are offered "***However, the tested β-lactams cannot be excluded as potential substrates, as in vitro expression of*** $bla_{OXA-1383}$ ***in P. litoralis may have been impaired by insufficient induction, while heterologous expression may have limited enzyme activity through inefficient protein folding and localisation***" (L362-365).

## Additional edits for the editor

A few minor edits have been applied to improve clarity and consistency:

Grammatical mistake at L320 has been amended.

Clarification that E30 refers to *P. litoralis* (L100).

Clarification in reference to “horizontal gene transfer ***of mcr-12***” (L288).

The *mcr-12* “*location*” at L333 has been made clearer to “***genomic** location*”.

Two instances where oxford commas were missing (L150, L427).

Hyphen replaced with en-dash (L520).

Supplementary Table 2, and Supplementary Table 5: header of the cured strain has been changed to “*P. litoralis* ***(pPLE30.2⁻)***” to be consistent with labelling in Figure 4.

Contents in tables have been centre justified.
